# Integrated Adaptive Responses of Rhizosphere Microbial Communities and Functional Genes to Cadmium Stress in Ryegrass (*Lolium perenne* L.)

**DOI:** 10.3390/plants15172634

**Published:** 2026-08-28

**Authors:** Bingjian Cui, Erping Cui, Rong Wu, Zhongyang Li, Xiangyang Fan, Chuncheng Liu

**Affiliations:** 1 Institute of Farmland Irrigation, Chinese Academy of Agricultural Sciences, Xinxiang 453002, China; cuibingjian@caas.cn (B.C.);; 2 Agriculture Water and Soil Environmental Field Research Station of Xinxiang, Chinese Academy of Agricultural Sciences, Xinxiang 453002, China; 3 Jiangxi Hydrological Monitoring Center, Nanchang 330002, China; 4 Key Laboratory of High-Efficient and Safe Utilization of Agriculture Water Re-Sources, Chinese Academy of Agricultural Sciences, Xinxiang 453002, China

**Keywords:** *Lolium perenne*, high-throughput sequencing, Cd toxicity, rhizosphere microbiome, functional genes

## Abstract

Heavy metal pollution impacts on soil microorganisms are of growing concern. This study employed high-throughput sequencing and qPCR to investigate rhizosphere microbial communities and functional genes responses in ryegrass (*Lolium perenne* L.) under cadmium (Cd) stress. Results showed dose-dependent shifts in microbial diversity, metabolic pathways and functional gene abundance. The relative abundances of bacterial classes Alphaproteobacteria and Bacteroidia and genera *Cellvibrio* and *Algoriphagus* increased with Cd levels, suggesting roles in Cd adaptation. The classes Actinobacteria, Saccharimonadia, and Acidimicrobiia and the genera *Rheinheimera*, *Pseudomonas*, and *Sphingomonas* decreased. The fungal genera *Acremonium*, unclassified Hypocreales, *Cladosporium* and *Paramyrothecium* increased under high Cd, while *Albifimbria*, *Fusarium*, and *Ramichloridium* decreased. Differential adaptive strategies to Cd toxicity were observed between bacterial and fungal phyla. Redundancy analysis identified pH, EC, TN, and Cd as key determinants of microbial variation. FAPROTAX prediction revealed significant changes in dominant functional groups involved in nutrient cycling, and FunGuild analysis indicated increased saprophytic fungi with elevating Cd levels, potentially enhancing detoxification. Functional genes related to nitrogen and carbon cycling also varied with Cd gradients. This study systematically elucidated the response of rhizosphere soil microbial communities and functional gene abundance to Cd stress, providing mechanistic insights for developing ryegrass tolerance strategies.

## 1. Introduction

Heavy metals (HMs) have been found in a wide variety of environmental matrices, including soil, water, air, animals and plants, even exposure to the human body. HMs pose significant risks due to their inherent toxicity, environmental persistence and capacity for bioaccumulation [1]. The rapid development of industrialization and urbanization has led to the aggravation of soil heavy metal pollution, presenting a significant challenge to soil remediation and reclamation efforts [2]. A review of heavy metal pollution rates in Chinese farmland soil showed that Cd was ranked as the highest polluting heavy metal with the ratio of 7.75% [3]. Soil Cd contamination originated from both natural and anthropogenic sources, including atmospheric deposition, irrigation water, fertilizers, pesticides, and industrial waste discharges [4,5]. Improper irrigation with wastewater or reclaimed water can also lead to heavy metal contamination of soil [6,7]. Rice grains grown in soils at high risk of Cd exceed food safety thresholds, primarily due to industrial or other anthropogenic activities [8]. An increase in Cd levels in the soil could negatively impact its composition and performance, ultimately leading to a degradation in soil quality [9]. It might also inhibit plant root growth and photosynthesis, reducing the quality and yield of agricultural products [10,11]. Additionally, the pathways of food chain and human exposure could potentially raise the risks to human health [12,13]. Phytoremediation is recognized as an environmentally friendly and cost-effective bioremediation method. Previous studies have demonstrated that plants such as *Solanum photeinocarpum* [14], *Solanum nigrum* [15], *Lolium perenne* L. [16], *Arabidopsis halleri*, and *Sedum alfredii* [17] exhibited excellent performance in terms of their ability to tolerate and enrich Cd.

As a potential candidate for phytoremediation, ryegrass (*Lolium perenne* L.) is recognized as one of the plant species with high Cd-accumulating capacity [18]. The Cd concentrations in the shoots and roots of ryegrass were closely related to the effective Cd concentration, and the transfer of Cd to ryegrass was negatively affected by pH and positively affected by organic matter [19]. Microbial tolerance to Cd was significantly higher in cultivated soils than in uncultivated soils. Furthermore, ryegrass Cd accumulation showed strong positive correlations with total/diethylenetriaminepentaacetic acid (DTPA)-extractable Cd in the soil, indicating that soil Cd pollution not only threatens ecosystem health but also increases the risks of food chain transfer [20]. Ryegrass effectively reduced total Cd levels in soil over an optimal bioremediation period of 60 days, affecting the bioavailability of Cd [21]. Low Cd concentrations promoted increases in soil urease activity and microbial biomass carbon content. However, Cd concentrations exceeding 20 mg·kg^−1^ caused irreparable harm to ryegrass and soil microorganisms [22]. The rhizosphere refers to the soil surrounding plant roots that is influenced by root activity, as a unique hot spot in the soil that stimulates soil microbial activity and nutrient cycling in the vicinity of the roots by releasing root exudates [23]. The viability and functional activity of rhizosphere microbial communities in high concentrations of heavy metals play crucial roles in the detoxification of contaminated soil. Numerous studies have investigated the response of rhizosphere-associated microorganisms to soil Cd contamination. Cd contamination had no significant effect on the rhizosphere fungal community but was the main driving factor for shifts in the rhizosphere bacterial community structure [24]. Most recently, the rhizosphere has become the focus of attention for regulating the persistence, mobility and bioavailability of soil pollutants through root exudates-mediated microbial activity and plant–microorganism symbiosis. The migration rate of Cd in the rhizosphere was higher than the uptake rate by plant roots, and the soil microbial community structure was significantly reassembled after phytoremediation [25]. The biodegradation of soil pollutants is synergistically regulated by a network of microbes–soil enzymes–plants. Future research integrating enzyme engineering, QS-based biosensors, artificial intelligence-driven modeling, and synthetic QS circuits is expected to further optimize bioremediation strategies for heavy metal-contaminated soils [26].

Long-term Cd pollution significantly altered bacterial community composition and reduced rhizosphere diversity, potentially disrupting the major plant–bacteria interactions [27]. Cd-contaminated soil microbes, including crucial taxa such as Proteobacteria, *Sulfuricella*, and *Thiobacillus*, represented a potential resource for exploring Cd-resistant bacteria and highlighted their important physiological roles in coping with Cd contamination [28]. High Cd concentrations exerted toxic effects on soil organisms by disrupting microbial communities, inhibiting enzymes, and damaging cellular structures, ultimately impairing soil ecosystem functioning. Several studies have elucidated the synergistic effects of materials, microorganisms, and plants on enhancing the remediation of heavy metal-contaminated soils. Although organic amendments temporarily alleviated Cd toxicity, their long-term efficacy was constrained by organic matter decomposition, which might release bound metals and altered soil pH, thereby complicating bioremediation [29]. Nanoparticle application strengthened the survival of plant growth-promoting bacteria (PGPB) in the rhizosphere, boosting soil enzyme activities (dehydrogenase, catalase, phosphatase, urease) and increasing antioxidant production to mitigate Cd-induced stress injury to ryegrass [30]. Humic acid (HA) could enhance the growth of perennial ryegrass and its Cd phytoextraction capacity by improving soil microenvironment (increasing SOM, AP, AK and AN levels) and enriching rhizosphere growth-promoting bacteria (e.g., *Pseudomonas*, *Steroidobacter*, *Phenylobacterium*, and *Caulobacter*) [31]. The biochar-immobilized *Bacillus* sp. successfully colonized the rhizosphere, increasing rhizosphere microorganisms and enzyme activity, which contributed to a decrease in Cd concentration in ryegrass [32]. Proteobacteria abundance positively correlated with heavy metal gradients, and increased *Rhizobium* abundance might be related to Cd detoxification via the efflux pump gene *czcA* [33].

Despite the critical role that rhizosphere microbes play in assisting host plants to cope with heavy metal contamination, their community dynamics and functional diversity in response to varying Cd levels remain poorly understood. Here, we employed high-throughput sequencing and quantitative PCR (qPCR) to investigate the effects of cadmium (Cd) on microbial communities and functional genes within the rhizosphere of ryegrass (*Lolium perenne* L.) grown in soils spiked with Cd at concentrations ranging from 0 to 500 mg·kg^−1^ soil. This study aims to systematically elucidate the dose-dependent responses of rhizosphere soil microbes and functional genes in ryegrass to varying Cd concentrations, and to decipher the assembly mechanisms of microbial assemblages driven by Cd gradients. Our findings provide valuable insights into the succession processes of rhizosphere microorganisms and facilitate the identification and exploration of Cd-resistant or -tolerant microbial resources associated with ryegrass under Cd-contaminated soils.

## 2. Results

### 2.1. Physicochemical Properties of Soil

The soil physiochemical properties are summarized in Table 1. Compared with C0, soil pH increased significantly (*p* < 0.05) with increasing soil Cd concentrations, while under C500 treatment it decreased. EC was significantly increased in soil treated with C500, but no significant difference was found in other treatments. Soil OM and TP contents were not affected by heavy metal. TN content decreased first and then increased. The total soil Cd content increased significantly following the soil Cd-spiking levels (*p* < 0.05).

### 2.2. Diversity of Microbial Community in the Cd-Spiked Soil

A total of 742,315 and 1,081,157 clean reads of bacterial and fungal communities were obtained in rhizosphere soil samples after removing low-quality reads and adaptor reads, respectively. The subsampled OTU tables were created by the minimum number of sequences in all samples from the original OTU table for downstream analysis; a total of 3338 and 831 OTUs were generated at 97% identity of bacterial and fungal communities, respectively. All bacterial OTUs were assigned to 35 phyla, 101 classes, 252 orders, 415 families, and 740 genera. For eukaryotes, there were 12 phyla, 35 classes, 80 orders, 176 families, and 320 genera represented.

The Chao1, Coverage, Shannon and Simpson indices were calculated to reflect α diversity of microbial communities (Table 2). For bacteria, the Chao1 and Coverage showed no significant differences among the treatments, while significant differences in soil bacterial community diversity (Shannon and Simpson indices) were observed across samples. Along with the increase in the Cd concentration, the Shannon index of bacteria in rhizosphere soil decreased significantly except for C500. The declining trend of the Shannon index along the Cd concentration gradient indicated ecological stress induced by Cd toxicity, which resulted in decreased community diversity and the dominance of a few tolerant taxa. The Simpson index increased significantly in the C25, C50, and C100 treatments, suggesting that bacterial community diversity was reduced under these Cd levels. The results showed no significant differences in community coverage and Shannon index of fungal community among different treatment groups. Unlike bacteria, the richness index of fungi in rhizosphere soil was significantly different among all treatments, and C50 and C100 were significantly higher than other treatments. The Simpson index of the fungal community under the C500 treatment was significantly higher than that of the other treatments, indicating a decrease in fungal community diversity. Taken together, these results indicated that bacteria exhibited higher sensitivity to Cd stress than fungi, and the shifts in community diversity indices under extreme Cd contamination (C500) were associated with the establishment of distinct community structures. The rarefaction curves were constructed based on the observed OTUs. It was observed that the rarefaction curves tended to be flat, and the amount of sequencing data reached saturation, suggesting that sequencing depth can cover most species in rhizosphere microbial communities (Figure S1).

**Figure 1:**
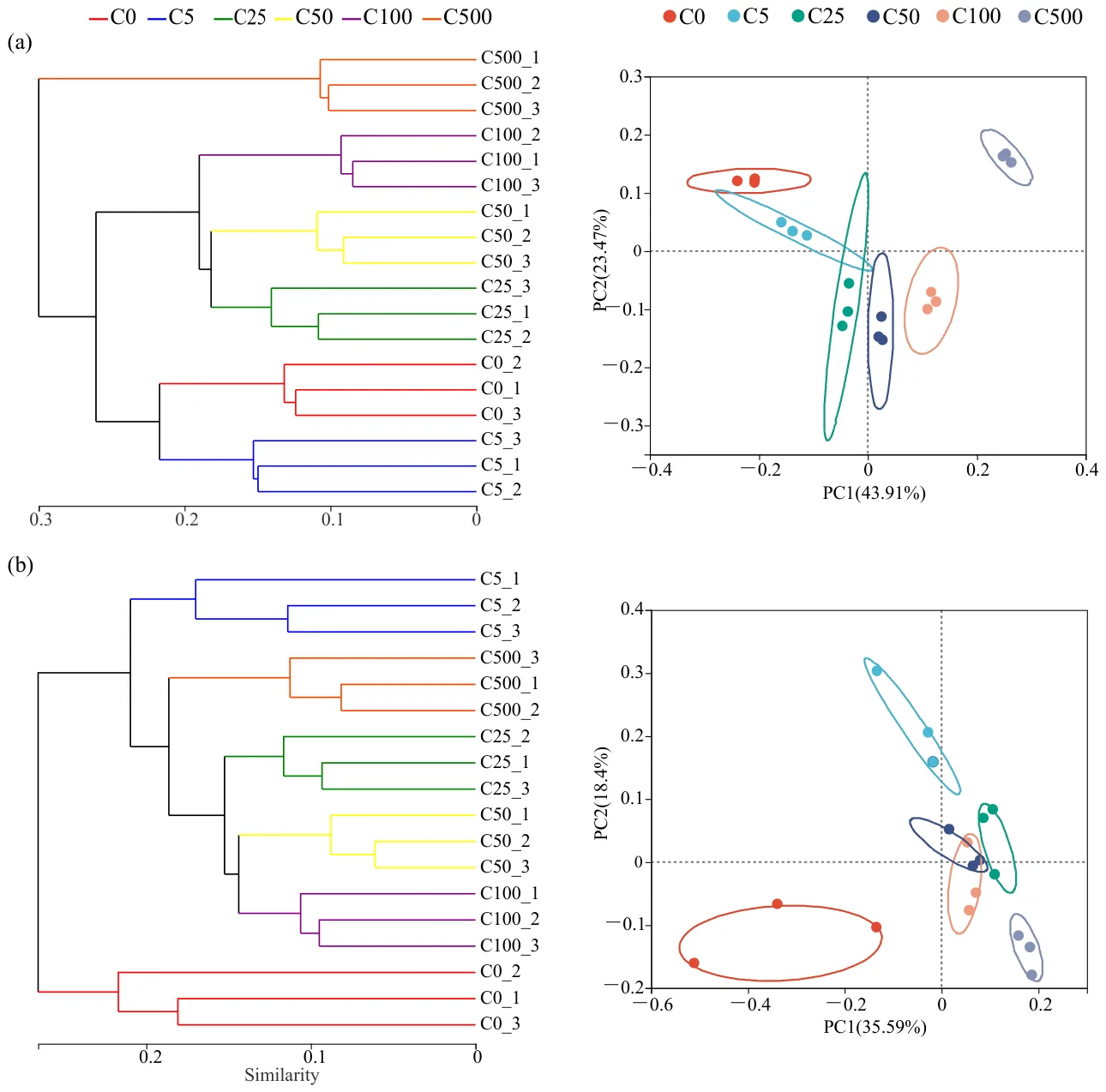
The hierarchical clustering and principal co-ordinates analysis (PCoA) of bacterial (**a**) and fungal (**b**) communities.

*β* diversity analysis was performed by hierarchical clustering and principal co-ordinates analysis (PCoA) based on Bray–Curtis distances at the OTU level. The hierarchical clustering result showed that the samples could be divided into three significantly different bacterial groups according to different distance thresholds, indicating that the bacterial community composition of the rhizosphere soil spiked with heavy metal was significantly different (Figure 1a). PCoA at the OTU level showed that soil bacterial community composition was significantly different under heavy metal stress (*R*^2^ = 0.9909, *p* = 0.0010) (Figure 1a). Fungal community in the rhizosphere soil was divided into two distinct groups, which were also influenced by heavy metal stress (Figure 1b). The Bray–Curtis distance was calculated to determine the similarity and dissimilarity among the different treatment groups. PCoA showed a significant separation of bacterial communities between the control and the other five levels of Cd-spiked soil, indicating that heavy metal stress had a significant effect on rhizosphere soil fungal communities (*R*^2^ = 0.7212, *p* = 0.0010) (Figure 1b). Permutational multivariate analysis of variance (PERMANOVA) indicated that microbial community composition was statistically different by soil physicochemical factors (pH, EC, OM, TN, TP, and Cd) (*p* = 0.001, *F* = 12.169 for prokaryote 16S and *F* = 7.637 for eukaryote ITS).

### 2.3. Microbial Community Composition of Different Cd-Treated Soils

Venn diagram analysis of the unique and shared core OTUs for bacterial and fungal communities under different Cd stress is shown in Figure S2. Bacteria accounted for a high proportion of shared OTUs (1057, 31.67%), and the unique OTUs were 92 (2.76%, C0), 69 (2.07%, C5), 34 (1.02%, C25), 31 (0.93%, C50), 36 (1.08%, C100), and 129 (3.86%, C500) (Figure S2a). C500 had the highest proportion of unique OTUs, indicating significant enrichment of core OTUs under C500 treatment. Venn diagram analysis revealed a total of 103 shared OTUs in the soil fungal community. The unique core OTUs for fungal communities of different treatment groups were 50 (C0), 35 (C5), 142 (C25), 51 (C50), 87 (C100), and 54 (C500), accounting for 6.02%, 4.21%, 17.09%, 6.14%, 10.47% and 6.50% of the total OTUs, respectively (Figure S2b). Unique OTUs of fungal communities were observed for each treatment group, indicating that the core OTUs were enriched to varying degrees across treatment groups. Cd stress shifted dominant bacterial taxa toward unclassified resistant species, which was consistent with an increase in unique OTUs observed in the Venn diagram.

The class-level taxonomic composition of rhizosphere bacterial communities under different treatments is shown in Figure S3a. The rhizosphere bacterial sequences were assigned to six dominant phyla (Proteobacteria, Bacteroidota, Actinobacteriota, Patescibacteria, Acidobacteriota, and Chloroflexi), which together comprised 89.56–91.54% of the total OTUs. At the class level, Gammaproteobacteria, Alphaproteobacteria, Bacteroidia, Actinobacteria, Acidimicrobiia, and Saccharimonadia were consistently predominant across all treatments, collectively accounting for >75% of the total bacterial community (Figure S3a). Additionally, some minor classes were also detected, including Vicinamibacteria, Blastocatellia, Anaerolineae, Chloroflexia, and Verrucomicrobiae. Although the bacterial community structure was similar among different treatments, variations in relative abundances were observed in individual treatment groups. Notably, the relative abundance of Bacteroidia increased with increasing Cd concentration, whereas those of Actinobacteria and Acidimicrobiia decreased. The relative abundance of Saccharimonadia was promoted by low Cd concentrations, but inhibited under high Cd stress. The fungal community was mainly composed of three dominant phyla, including Ascomycota, Glomeromycota, and Basidiomycota, which accounted for >97%. At the class level, Sordariomycetes, Glomeromycetes, and Dothideomycetes were the dominant taxa, while Eurotiomycetes, Leotiomycetes, and unclassified Mortierellomycota exhibited low relative abundance (Figure S3b). The relative abundances of Glomeromycetes and Mortierellomycota decreased significantly under the C500 treatment, whereas that of Sordariomycetes increased.

**Figure 2:**
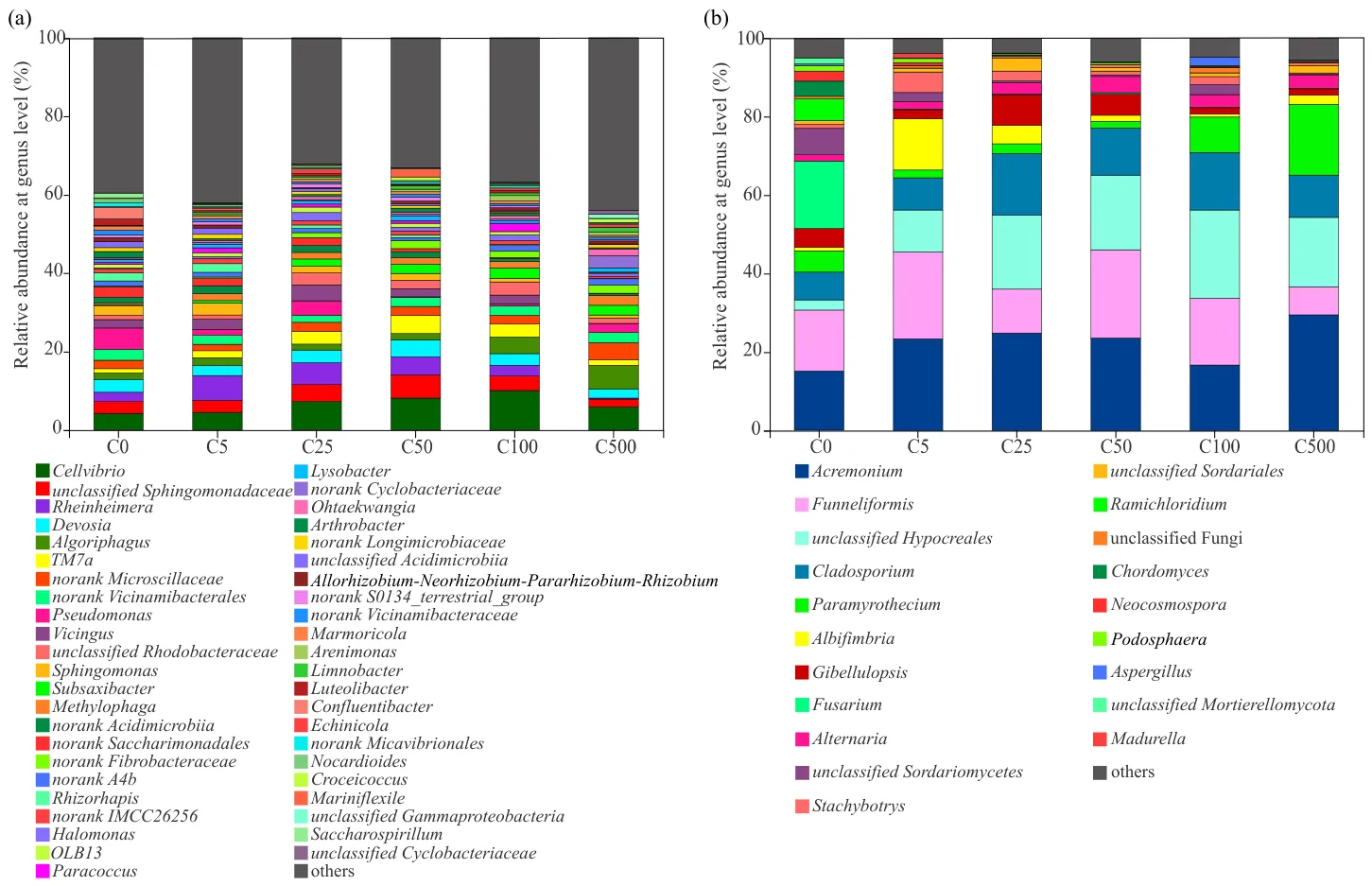
Relative abundance (%) of bacteria (**a**) and fungi (**b**) at genus level in rhizosphere soils under different Cd treatments.

A total of 46 dominant bacterial genera and 20 dominant fungal genera (>1% relative abundance) were identified across all treatments (Figure 2). While the bacterial community composition remained similar among all treatments, significant differences in the relative abundances were observed (Figure 2a). The relative abundances of *Cellvibrio*, *unclassified Sphingomonadaceae*, *Rheinheimera*, Vicingus, *Sphingomonas*, and *Paracoccus* showed a trend of increasing first and then decreasing with the increase in heavy metal concentrations. The relative abundances of *Algoriphagus*, *Subsaxibacter*, *Methylophaga*, and *unclassified Cyclobacteriaceae* in the C500 treatment were significantly increased compared with that in the C0 treatment, while *Pseudomonas*, *Arthrobacter*, *Marmooricola*, *Nocardioides*, and *Saccharospirillum* decreased significantly. Compared with the C0 treatment, the C500 treatment induced a marked increase in the relative abundances of *Acremonium*, *unclassified Hypocreales*, *Cladosporium*, *Paramyrothecium*, *Albifimbria*, and *Alternaria* (Figure 2b). The relative abundances of *Funneliformis*, *Gibellulopsis*, and *Fusarium* varied significantly across Cd concentrations. However, it was observed that high levels of Cd in the treatment resulted in a decrease in the relative abundance of these fungal genera. The microbial communities of rhizosphere soil samples treated with different Cd were detected for significant differences between groups; the results showed significant differences among the top 15 abundant bacterial and fungal genera (Figure S4). The significant differences observed among the groups at the genus level were mainly attributed to the presence of Proteobacteria (bacteria) and Ascomycota (fungi).

LefSe analysis was performed to identify biomarker taxa specific to each Cd treatment. In the C0 soil, *Pseudomonas*, *norank Saccharimonadales*, *Luteolibacter*, *Arthrobacter*, *Saccharospirillum*, and *Nocardioides* were significantly enriched. In the C5 soil, *Rheinheimera*, *Sphingomonas*, *Rhizorhapis*, *Thalassospira*, and *Noviherbaspirillum* were significantly enriched. In the C25 soil, *Vicingus*, *Halomonas*, *Echinicola*, and *Erythrobacter* were significantly enriched. In the C50 soil, *unclassified Sphingomonadaceae*, *TM7a*, *Flavobacterium*, and *Salinirepens* were significantly enriched. In the C100 soil, *Cellvibrio*, unclassified Rhodobacteraceae, *Subsaxibacter*, *Paracoccus* and *Arenimonas* were significantly enriched. In the C500 soil, the enriched taxa included *Algoriphagus*, *norank Cyclobacteriaceae*, *Methylophaga*, *Ohtaekwangia*, and *Limnobacter*. For fungal community, the taxa *Fusarium*, *unclassified Sordariomycetes*, *Chordomyces*, and *Neocosmospora* were significantly enriched in the C0 soil. *Albifimbria*, *Stachybotrys*, *Schizothecium* and *Emericellopsis* were significantly enriched in the C5 soil. *Unclassified Glomerellales*, *Diversispora*, and *Rhizophagus* were significantly enriched in the C25 soil. *Funneliformis*, *Alternaria*, and *Mortierella* were significantly enriched in the C50 soil. Unclassified Hypocreales, *Aspergillus* and unclassified Ascomycota were significantly enriched in the C100 soil. *Paramyrothecium*, *unclassified Rozellomycota*, *unclassified Chytridiomycota*, and *Pseudeurotium* were significantly enriched in the C500 soil (Figure S5a,b). These results revealed distinct biomarker profiles between bacterial and fungal communities at the genus level in various Cd treatment scenarios.

### 2.4. Correlation Between Environmental Parameters and Microbial Communities

**Figure 3:**
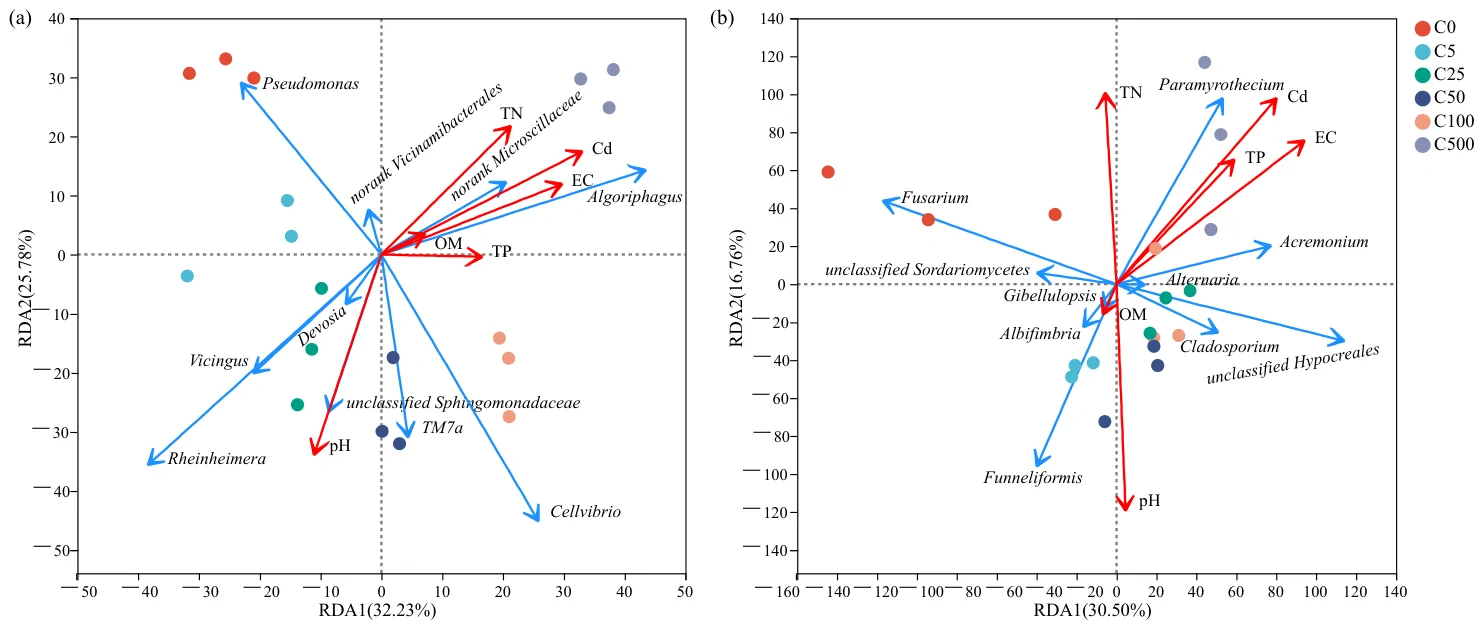
Redundancy analysis (RDA) of bacterial (**a**) and fungal (**b**) communities for the soil environmental factors; the top 10 genera are shown in the figures. Abbreviations: EC: electrical conductivity; OM: organic matter; TN: total nitrogen; TP: total phosphorus; Cd: cadmium.

Redundancy analysis (RDA) was performed to explore the correlation between the microbial communities and soil physicochemical factors (pH, EC, OM, TN, TP, and Cd). For bacterial communities, pH, EC, TN, and Cd were significantly correlated with community changes at the genus level (Figure 3a). For fungal communities, pH, EC, TN, TP, and Cd were significantly correlated with community changes at the genus level (Figure 3b). The RDA showed that the soil physicochemical properties explained 58.01% (RAD1 32.23% and RDA2 25.78%) and 47.26% (RAD1 30.50% and RDA2 16.76%) of the variances in bacterial and fungal community at the genus level, indicating that these environmental factors were important driving factors affecting the diversity and composition of the rhizosphere soil microbial community.

**Figure 4:**
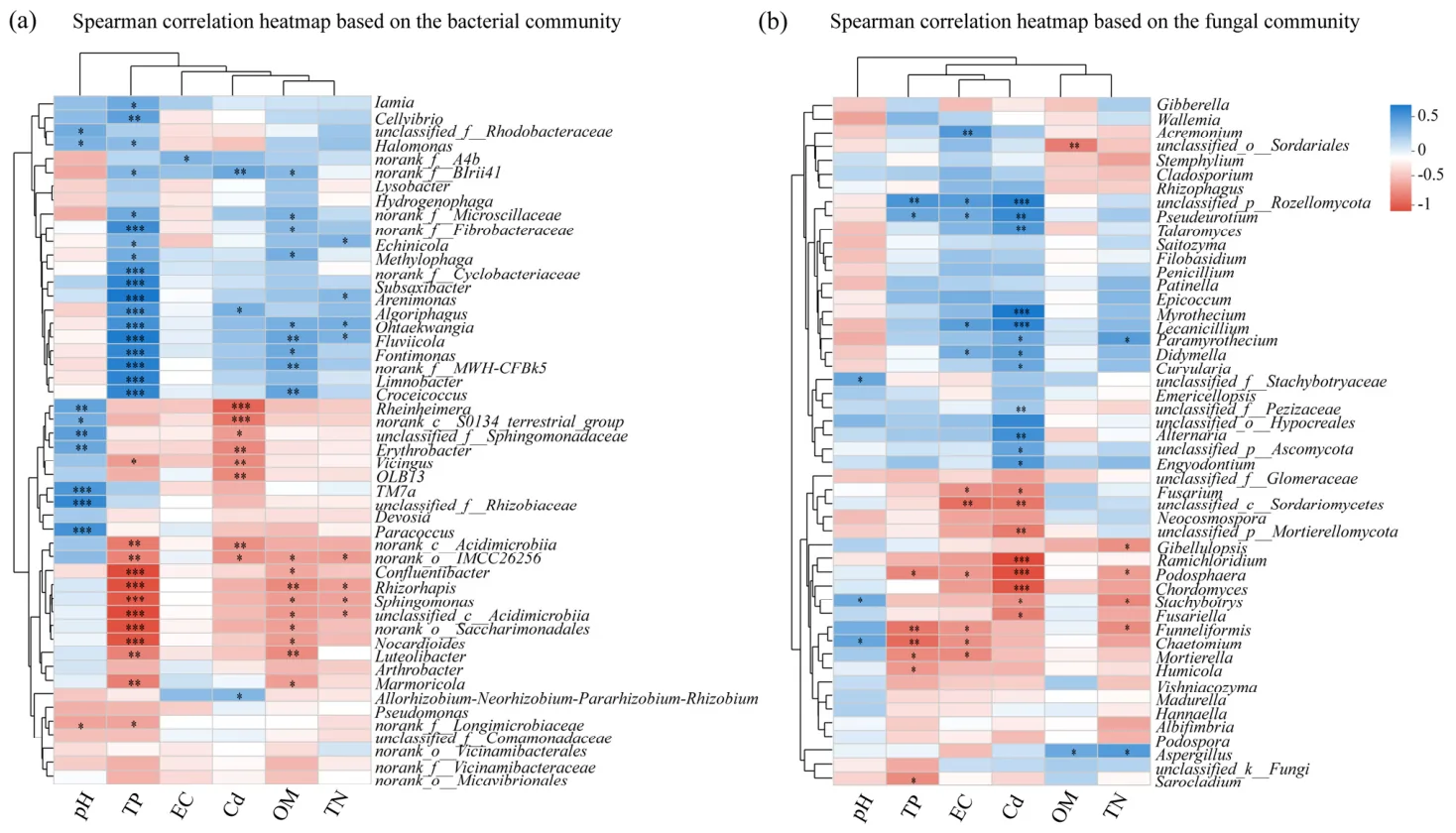
The Spearman correlation analysis between the soil environmental factors and the relative abundance of bacterial (**a**) and fungal (**b**) communities at the genus levels. Legend on the right side shows the colors of different *p* value ranges (* 0.01 < *p* ≤ 0.05; ** 0.001 < *p* ≤ 0.01; *** *p* ≤ 0.001). Abbreviations: EC: electrical conductivity; OM: organic matter; TN: total nitrogen; TP: total phosphorus; Cd: cadmium.

Spearman correlation heatmaps were generated to assess the relationship environmental variables and the community composition of the top 50 bacterial and fungal genera (Figure 4). Heatmap analysis of the correlation between dominant bacterial genera and environmental factors shows that environmental factors are significantly positively correlated with the abundance of 28 dominant bacterial genera, and significantly negatively correlated with the abundance of 17 dominant bacterial genera (*p* < 0.05) (Figure 4a). pH, EC, TP, TN, and Cd were significantly correlated with the rhizosphere bacterial community. A strong positive correlation was observed between environmental factors and the abundance of 17 dominant fungal genera, while 15 dominant fungal genera showed significant negative correlations (*p* < 0.05) (Figure 4b). The rhizosphere fungal community had a significant association with EC, TP, TN, and Cd. The Mantel test analysis demonstrated that EC, TN, TP, and Cd all played a significant role in shaping the structure of bacterial and fungal communities (*r* = 0.6845 and *p* = 0.0010 for bacteria, *r* = 0.2973 and *p* = 0.050 for fungi). It should be noted that the Mantel test identified correlations between environmental factors and community structure rather than direct causal or ecological interactions.

### 2.5. Network Analysis and Predictive Functional Analysis

**Figure 5:**
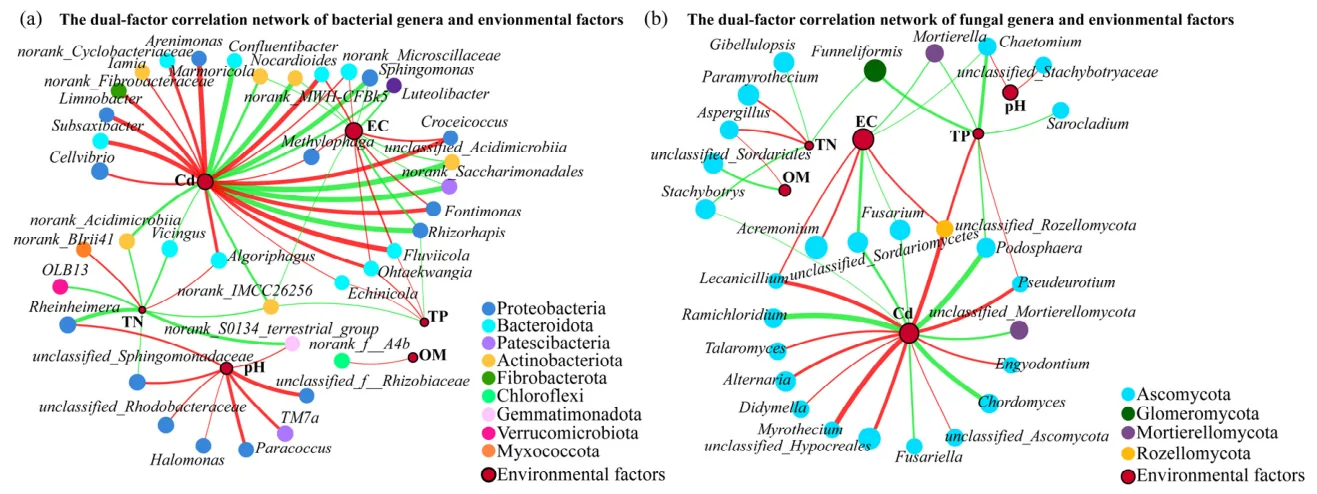
Network analysis applied to the bacterial (**a**) and fungal (**b**) genera. The size of nodes represented species abundance. The red line represented positive correlation, and the green line represented negative correlation. Abbreviations: EC: electrical conductivity; OM: organic matter; TN: total nitrogen; TP: total phosphorus; Cd: cadmium.

The top 50 most abundant genera were selected to determine the interactions between rhizosphere microbiota and environmental factors by Spearman correlation analysis. As revealed by the edge numbers and nodes, the bacterial genera exhibited greater complexity and connectivity with environmental factors than those of the fungal genera. Cd exhibited stronger co-occurrence patterns with key genera in both rhizosphere bacterial and fungal communities (Figure 5). Additionally, bacteria genera displayed 37 positive and 28 negative links to environmental factors, while fungal genera showed 20 positive and 21 negative links with these same factors.

Functional Annotation of Prokaryotic Taxa (FAPROTAX) analysis identified 42 functional groups associated with established metabolic and ecological functions (Figure S6). Chemoheterotrophy and aerobic chemoheterotrophy were the most abundant functions in all treatments. Functions related to the carbon and nitrogen cycling were also dominant in each treatment, with their relative abundances varying with Cd concentration. The top 15 functional groups are shown in Figure S7. Among these, 12 functional groups showed statistically significant differences among the treatments, as determined by the Kruskal–Wallis H test. The relative abundances of dominant functional groups associated with methylotrophy, methanol oxidation, and nitrate reduction increased significantly under Cd treatment (*p* < 0.05), whereas those related to nitrate respiration, nitrogen respiration, nitrite respiration, and nitrite denitrification decreased significantly under high Cd concentration (C500) (*p* < 0.05). BugBase phenotypic prediction revealed that Cd treatment significantly increased the levels of Gram-negative, potentially pathogenic, and facultatively anaerobic bacteria, while biofilm-forming and Gram-positive bacteria decreased significantly (Figure S8). Gram-negative bacteria were significantly enriched in rhizosphere soil under Cd stress. FUNGuild was applied to annotate functional classification of fungal OTUs. A total of 63 functional groups were identified, among which those with relative abundances > 1% were retained for further analysis. The fungal communities were categorized as saprotroph, pathotroph, and symbiotroph based on their trophic mode, accounting for more than 90% (Figure S9a). The relative abundance of saprophytic type was significantly higher in Cd-treated rhizosphere soils than in the control (*p* < 0.05). The lowest abundances of symbiotrophic (*Arbuscular Mycorrhizal*) and pathotrophic (plant and animal pathogen) types were observed in the C500 group. Treatment with Cd is clearly distinguished from treatment without Cd, and the relative abundance of the pathological-saprobic-Symbiotic trophic type in C0 was the highest (Figure S9b).

### 2.6. qPCR Analysis of Functional Genes

**Figure 6:**
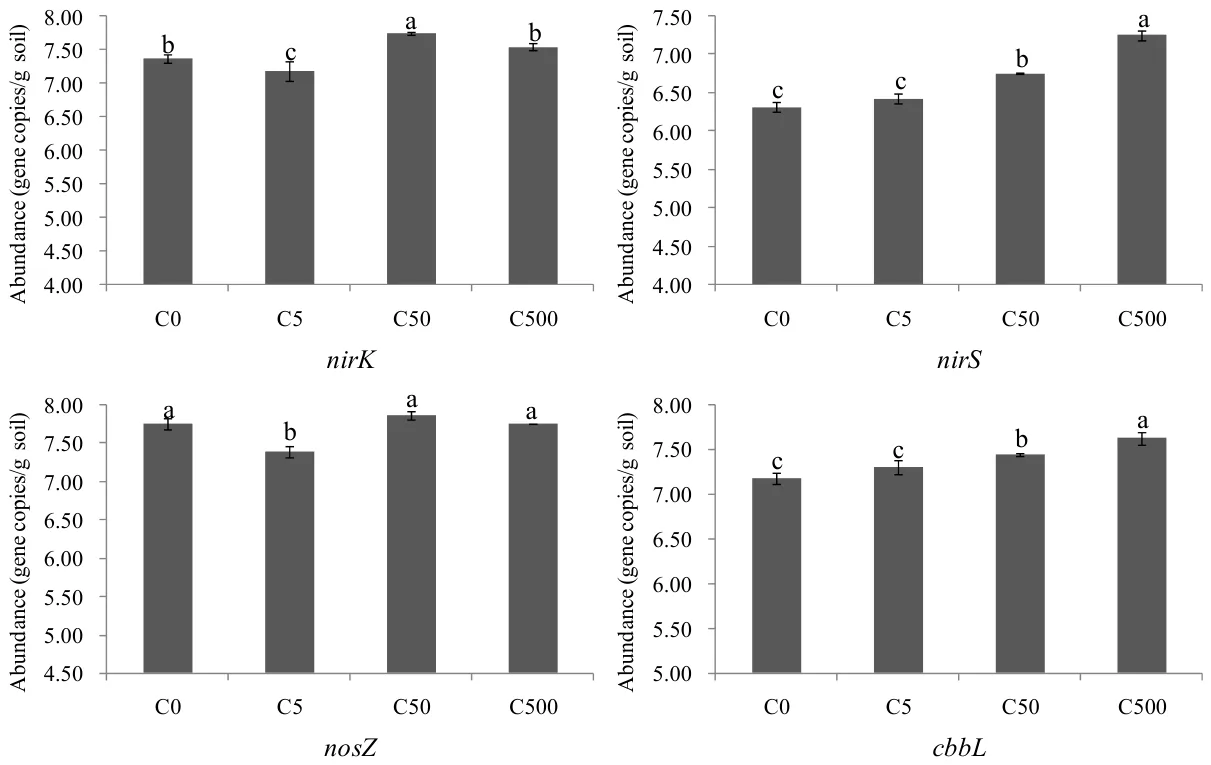
Variation in functional gene abundance in rhizosphere soil under different Cd concentrations.

In this study, qPCR was used to determine the abundances of N- and C-related functional genes (*nirK*, *nirS*, *nosZ*, and *cbbL*) in rhizosphere soil. Cd had a significant effect on the abundances of these functional genes. The absolute abundances of functional genes in rhizosphere soil were 1.59 × 10^7^–5.72 × 10^7^ (*nirK*), 2.11 × 10^6^–1.81 × 10^7^ (*nirS*), 2.49 × 10^7^–7.43 × 10^7^ (*nosZ*), and 1.52 × 10^6^–4.26 × 10^7^ (*cbbL*) copies per gram, respectively (Figure 6). The absolute abundance of denitrifying genes *nirK* and *nosZ* decreased significantly only in C5 (*p* < 0.05). Compared with the control (C0), the absolute abundance of the denitrifying gene *nirK* significantly increased by 58.02% in the C50 treatment, while *nosZ* abundance decreased by 56.55% in C5 (*p* < 0.05). In contrast, with increasing Cd concentration, the abundance of *nirS* and *cbbL* genes gradually and significantly increased (*p* < 0.05), reaching the highest levels in the C500 treatment, representing 7.55-fold and 1.80-fold increases over the control, respectively. Pearson correlation analysis between soil properties (pH, EC, OM, TN, TP, and Cd) and functional gene abundances (*nirS*, *nirK*, *nosZ*, and *cbbL*) revealed that *nirS* and *cbbL* abundances were significantly positively correlated with EC (*p* < 0.01) and Cd (*p* < 0.01). pH was significantly negatively correlated with TN, TP, and Cd, while EC was strongly related to TN and Cd (*p* < 0.05). TN and TP were positively correlated with Cd and TN, respectively (*p* < 0.05). In addition, significantly positive correlations were observed between *nirK* and *nosZ*, and between *nirS* and *cbbL* (*p* < 0.01) (Table S2).

## 3. Discussion

### 3.1. Response of Rhizosphere Microbial Community Structure and Diversity to Cd-Spiked Soil

Heavy metal pollution has a significant impact on soil microbes, mainly as follows: (1) heavy metal in soil has an important impact on microbial biomass, soil basal respiration and soil enzyme activity [34,35]; (2) microorganisms can affect the availability of heavy metals in soil through adsorption, transformation, metabolic activity [36]. Heavy metal pollution could decrease the richness of microbial community in soil [37]. The research revealed that Cd contamination could potentially reduce microbial diversity and further alter the community structure in the soil [28]. No significant differences in bacterial richness were observed among all treatments, whereas fungal richness exhibited a trend of first increasing and then decreasing. Both bacterial and fungal community diversity decreased significantly under Cd stress. Plant rhizosphere and endosphere harbored diverse bacteria and fungi, some of which have been demonstrated to promote plant growth and regulate metal uptake in heavy metal-contaminated soils [38]. Environmental selection pressure drives a shift from non-adapted to adapted microorganisms, leading to changes in microbial community composition. Under heavy metal stress, microorganisms are categorized into two types: sensitive (unable to survive) and resistant (capable of surviving). Additionally, certain microorganisms defined as “actors” mitigate heavy metal stress by immobilizing or accumulating heavy metals, thereby facilitating the survival of other microbes [39]. Heavy metal contamination could induce shifts in soil microbial community structure, often favoring the dominance of metal-tolerant microbial groups [40]. Cd significantly altered bacterial communities without affecting fungal communities or reducing 16S rRNA copy numbers [24]. In this study, Cd pollution altered the composition and structure of the rhizosphere soil microbial community, and significantly affected the α-diversity of both bacterial and fungal communities in ryegrass. Overall, these results indicated that Cd exposure reshaped the community structure and functional diversity of the rhizosphere microbiome of ryegrass. Typically, heavy metal pollution could result in a decrease in microbial diversity, and enrichment of tolerant taxa through the environmental filtering and adaptive processes. This might be partly attributed to the buffering capacity of the host plant, which could mitigate Cd toxicity in the rhizosphere soil.

Soil microbial community structure and composition analysis provided insights into the differential response of bacteria and fungi to increasing Cd concentrations. Heavy metal pollution significantly reduced the diversity of rhizosphere soil microbial communities. The relative abundances of Actinobacteriota (Actinobacteria and Acidimicrobiia), Patescibacteria (Saccharimonadia), Acidobacteriota (Vicinamibacteria and Blastocatellia), and Chloroflexi (Anaerolineae and Chloroflexia) decreased under Cd stress. Soil bacteria were more sensitive than fungi to the Cd stress. A significant increase in the relative abundance of Proteobacteria and Bacteroidetes was observed in Cd-contaminated rhizosphere soils, exhibiting high tolerance capacity against Cd. Studies have shown that the relative abundance of Proteobacteria, Bacteroidetes, and Firmicutes is highest in Cd-contaminated soils [41]. Cd at different levels was significantly correlated with the diversity and composition of bacterial communities in the rhizosphere. A total of 10 mg·L^−1^ Cd (II) reduced microbial diversity and altered the whole microbial community structure [42]. Soil bacterial α diversity declined significantly at higher Cd concentration. The relative abundances of *Rheinheimera*, *Pseudomonas*, *Sphingomonas*, *Arthrobacter*, *Marmoricola*, *Nocardioides*, and *Saccharospirillum* decreased significantly as the Cd levels increased. This is consistent with field research by Song et al. [27]. The presence of 20 mg·kg^−1^ Cd in the soil resulted in the highest relative bacterial abundance, bacterial community diversity and carbon utilization diversity, and the relative abundance of *Pseudomonas* was significantly higher in the heavy metal-treated soils [43]. Heavy metal stress not only adversely affects the physicochemical properties of agricultural soils, but also alters the diversity, composition, and activity of soil microbial communities. The proportion of heavy metal-immobilizing bacteria, including *Brevundimonas*, *Serratia*, *Arthrobacter*, and *Pseudarthrobacter*, was increased significantly in rhizosphere soil polluted by high concentrations of heavy metals, leading to a significant decrease in the contents of Cd and Pb in vegetable roots and above-ground parts [44]. Exposure to heavy metal stress resulted in a decrease in the abundance of both bacteria and fungi, and fungi were more sensitive than bacteria to heavy metal co-pollution [45]. The relative abundance of heavy metal-sensitive bacterial genera (e.g., *Pseudomonas*, *TM7a*, *Vicingus*, *Sphingomonas*, and *Rhizorhapis*) decreased, while heavy metal-tolerant or detoxifying bacterial genera (e.g., *Cellvibrio*, *Algoriphagus*, *Subsaxibacter*, and *Methylophaga*) increased in abundance through selective enrichment. It is speculated that the increase in adaptive microbial communities may enhance plant tolerance to heavy metals by secreting exopolysaccharides to precipitate heavy metal ions [46], altering cell membrane permeability [47], or engaging in plant symbiosis [48].

The soil fungal community was dominated by Ascomycota (Sordariomycetes, Dothideomycetes, Eurotiomycetes, and Leotiomycetes) and Glomeromycota (Glomeromycetes). At the class level, the relative abundances of Sordariomycetes and Dothideomycetes increased with increasing Cd concentrations, whereas that of Glomeromycetes decreased. Heavy metal pollution typically reduced the relative abundance of sensitive fungal taxa while promoting tolerant taxa through habitat adaptation, ultimately reshaping the fungal community structure [49]. The phylum Proteobacteria is the dominant bacterial phylum encountered in the rhizosphere, but fungi such as those in the phyla Ascomycota and Glomeromycota are also an integral component of the rhizosphere microbiome [50]. Typically, heavy metal pollution could decrease the microbial diversity and enrich tolerant species. Chen et al. (2014) [51] reported that long-term heavy metal pollution decreased significantly the abundance and diversity of fungi in a rice paddy soil. Fungal α diversity in rhizosphere soil was more affected by high Cd concentration, resulting in a significantly higher Simpson index for C500 compared to the other treatments. Soil heavy metals could affect the functional diversity of fungi, leading to changes in fungal community structure, which in turn exerts selective pressure on soil fungi [52]. Other studies indicated that soil fungi adapted to varying levels of heavy metal pollution by altering community structure [53]. The soil with high HM levels showed decreased abundances of *Aspergillus*, *Fusarium*, *Podospora*, and *Gibberella*, while *Alternaria*, *Phoma*, and *Exophiala* increased significantly [54]. This study found that with increasing HM levels, the relative abundances of *Acremonium*, *unclassified Hypocreales*, *Cladosporium*, and *Paramyrothecium* significantly increased, while the relative abundances of *Alternaria*, *Albifimbria*, *Gibellulopsis*, and *Fusarium* significantly decreased. The decline in α-diversity under high Cd stress reflected the loss of sensitive taxa, accompanied by the enrichment of metal-tolerant taxa. This compositional shift might partially compensate for the loss of sensitive species and sustain essential functions (C and N cycling), emphasizing the need to integrate diversity and composition metrics for a comprehensive understanding of microbial responses to heavy metal stress.

### 3.2. Environmental Factors Affecting Microbial Community Diversity

RDA revealed the correlations between rhizosphere soil microbial community composition at the genus level under different treatments and environmental factors. The composition and structure of rhizosphere soil bacterial and fungal communities are significantly altered under high Cd concentrations. At the genus taxonomic level, environmental factors exhibited differential correlation patterns with bacterial and fungal community structures under Cd stress. Soil pH, EC, TN, and Cd were the most strongly correlated factors with the microbial community, suggesting their important roles in community assembly. The alkaline pH shifts might synergistically promote Cd immobilization through carbonate/hydroxide precipitation and adsorption processes, potentially influencing microbial community structure—a relationship that warranted further investigation. Notably, unlike traditional nutrient factors, Cd acts as a toxic environmental stressor to microorganisms. Mantel test results identified Cd and Zn as the primary drivers of microbial functional shifts, whereas the overall community structure and function were shaped by the combined effects of multiple heavy metals/metalloids and soil properties [55]. The rhizosphere exhibited significantly higher activities of C-, N-, and P-acquiring enzymes than bulk soil, indicating elevated microbial metabolic activity in response to HM-induced shifts in nutrient content and stoichiometry [56]. Spearman correlation analysis identified pH, EC, TN, and Cd as the key physicochemical parameters of rhizosphere soil, exhibiting the strongest correlation with changes in the relative abundance of microbial community. Genera including *Gaiella*, *Haliangium*, *Iamia*, *Novosphingobium*, *Pedomicrobium*, *Arenimonas*, *Bradyrhizobium*, and *Fodinicola* were significantly and positively correlated with soil available Cd content [57]. In this study, bacterial genera strongly correlated with soil physicochemical properties primarily belonged to the phyla Proteobacteria and Bacteroidetes, whereas the main fungal genera were affiliated with Ascomycota and Glomeromycota. The diversity of the rhizosphere microbial community is dynamically shaped by the physicochemical characteristics of the rhizosphere soil. Dominant fungal taxa exhibited higher sensitivity to different levels of Cd contamination compared to their bacterial counterparts. In summary, the Cd concentration gradients exerted strong environmental filtering on microbial communities through direct toxicity and indirect alterations of the rhizosphere environment, thereby driving complex community succession and potentially impairing soil ecological functions.

### 3.3. Functional Potentials Associated with the Soil Microbial Community

Heavy metal treatment not only significantly altered the structure and diversity of rhizosphere microbial communities but also influenced their functional metabolic pathways and phenotypic differences. The functional predictions derived from FAPROTAX and BugBase provide important insights into the potential mechanisms underlying microbial adaptation to Cd stress. FAPROTAX functional prediction analysis indicated that rhizosphere bacteria are primarily associated with chemoheterotrophy and aerobic chemoheterotrophy, as well as carbon- and nitrogen-cycling functions. The core functions of soil bacterial communities were metabolism and genetic information processing. With increasing heavy metal pollution, pathways such as the immune system, endocrine system, and secondary metabolite biosynthesis in bacterial communities were activated [27]. The variation in functional groups of rhizosphere bacterial communities might be closely related to the sensitivity of rhizosphere microorganisms to heavy metal stress and the enrichment of characteristic functional microorganisms. The loss of rare soil species reduced the α-diversity of rhizosphere microorganisms and altered their community structure and functional potential. Meanwhile, species co-occurrence network analysis revealed that protist and bacterial diversity, as well as protist–fungal network connectivity, most significantly contribute to plant Cd uptake and growth [58]. According to this study, heavy metal selective pressure determined the functional roles of microorganisms. FAPROTAX prediction indicated that the rhizosphere bacterial community in uncontaminated soil had stronger chemoheterotrophic potential, while the rhizosphere soil under Cd treatments showed higher functional potential for methylotrophy, methanol oxidation, nitrate reduction, fermentation and hydrocarbon degradation. Specifically, the enrichment of carbon and nitrogen functions under high Cd treatments suggested that hydrocarbon and nitrogen metabolism facilitated nutrient cycling and heavy metal detoxification by reducing the mobility and bioavailability of Cd.

The relative abundance of certain dominant KEGG pathways increased in Cd-contaminated soils, primarily involving the metabolism, biosynthesis and degradation of amino acids, fatty acids and nucleotides related to Cd tolerance in microorganisms [28]. Metagenomic analysis based on KEGG pathways identified 19 significantly altered metabolic pathway groups under combined microplastic and Cd pollution, including microbial metabolism and methane metabolism [59]. Predicted genes and proteins revealed remarkable metabolic diversity in microbes, highlighting their potential to oxidize, reduce, and remediate metals in heavy metal-contaminated soil [60]. The predicted functions of the rhizosphere microbiome were shaped by differential uptake of Cd and mineral elements, including sulfur oxidation, the formation of sulfur derivatives, human or plant pathogens, as well as functions related to iron oxidation and nitrate reduction [61]. KEGG pathway prediction suggested that although the microbial metabolism and proliferation were inhibited, effective Cd immobilization occurred in rhizosphere soil via cellular accumulation and biofilm sequestration induced by *Bacillus* bioaugmentation [62]. BugBase analysis revealed six out of nine predicted microbial phenotypes that exhibited significant differences under Cd stress. The relative abundances of stress-tolerant, potentially pathogenic, and Gram-negative bacteria significantly increased at high Cd level, while biofilm-forming and Gram-positive bacteria significantly decreased. The rhizosphere soil bacterial phenotypes of ryegrass were strongly influenced by *Bacillus* inoculation, with significantly increased relative abundance of genes related to facultatively anaerobic, biofilm-forming, potentially pathogenic and stress tolerant bacteria [62]. FUNGuild prediction identified saprotroph, arbuscular mycorrhizal, and plant pathogen as the dominant fungal functional groups. With increasing Cd concentration, the relative abundance of saprotrophic fungi significantly increased, whereas the plant pathogen group significantly decreased, suggesting a Cd-driven functional shift toward saprotrophic dominance. The dominant saprotrophic taxa (Sortariomycetes, Eurotiomycetes, and Dothideomycetes) have been previously reported in Cd-contaminated soils, reinforcing the ecological relevance of this functional transition [63].

### 3.4. Abundance of Functional Genes

In this study, the abundances of functional genes involved in denitrification (*nirK*, *nirS*, and *nosZ*) and carbon dioxide fixation (*cbbL*) were investigated in rhizosphere under different Cd treatments. Cd affected soil biogeochemical processes by altering microbial biomass and activity, while soil total carbon and nitrogen have been reported to increase significantly under high Cd conditions [64]. The abundances of *nirK*, *nirS*, and *cbbL* in rhizosphere soil were sensitive to Cd pollution. Quantitative results indicated that the selected genes involved in carbon and nitrogen cycling exhibited a certain degree of tolerance to heavy metal stress. Nitrifying genes (*narG*, *nirK*, *nirS*, and *nosZ*) were mainly related to water-soluble organic carbon (WSC) and soil nitrite reductase activity (S-NiR), whereas the denitrifying gene (*amoA*) was associated with pH, electrical conductivity, NO_3_^−^, and S-NiR [65]. The abundances of *nirS* and *cbbL* were significantly positively correlated with soil electrical conductivity (EC) and Cd levels. The abundance of ammonia-oxidizing and denitrifying microorganisms was significantly correlated with soil pH and exchangeable Cd [66]. Besides plants, exogenously introduced microbes can also influence functional gene responses to heavy metal pollution. *Bacillus* inoculation has been shown to reduce heavy metal bioavailability, thereby increasing *AOA* and *AOB* abundances under varying Cd levels [41]. *Cenococcum geophilum* alleviated Cd stress by enhancing the absorption and assimilation of inorganic nitrogen in plants and inhibiting Cd transport from roots to shoots [67]. Inoculation with rhizobium in Cd-contaminated soil altered the composition and functional gene abundance of the rhizosphere microbial community, leading to increased *nifH* abundance but decreased abundances of the *amoA*, *amoB*, *nirS*, *nirK*, and *nosZ* [68]. A study on water management in paddy soils under heavy metal pollution demonstrated that Cd contamination significantly reduced the copy numbers of *nirS*, *nirK*, and *nosZ*, with high Cd level being more toxic to nitrifying and denitrifying bacteria under non-flooded condition, thereby suppressing the nitrogen transformation processes mediated by microorganisms [69]. The response of functional gene abundances to Cd contamination varies depending on Cd concentration and soil conditions. Cd levels below 10 mg·kg^−1^ increased the abundance of the *nirS* gene in alkaline soils, while decreasing the abundances of Cd-sensitive *nosZ*, *norB*, and *nirK* genes [70]. However, with increasing Cd pollution levels, the abundances of denitrification genes (*nirK*, *nirS*, and *nosZ*) in rhizosphere soil increased, and low Cd concentration even resulted in higher abundances of key denitrifying genes (*narG*, *nirS*, *norB*, *napA*, *nirK*, and *nosZ*) [71]. These inconsistent findings suggested that the direction and magnitude of Cd-induced changes in functional gene abundance were highly context-dependent, potentially modulated by soil properties, microbial community, and Cd bioavailability. Cd generally inhibited soil microbial biomass, thereby impeding carbon and nitrogen cycling and consequently reducing soil nitrogen availability [72]. Cd altered microbial C/N metabolism from catabolic to anabolic dominance, indicating nutrient-based adjustments in microbial N-cycling to enhance metal resistance and microbial adaptation [73]. Heavy metal concentrations affected carbon cycle genes, and the copy numbers of these genes increased to varying extents under heavy metal stress. Long-term Pb and Cd contamination resulted in higher abundances of carbon cycle genes compared with the control, suggesting that heavy metals might promote certain steps of the soil carbon cycle [74]. Similarly, Cd-containing treatments altered the abundances of nitrogen- and phosphorus-cycling genes in the sorghum rhizosphere soil [59]. Elevated heavy metal concentrations significantly intensified microbial carbon (C) limitation. The energy metabolism adopted by microorganisms under long-term heavy metal stress might accelerate soil C release, ultimately constraining soil C sequestration [75]. The present study has several limitations, primarily due to its focus on the abundance changes of select functional genes rather than their actual expression or activity. Numerous critical issues remain inadequately addressed. For example, how the high-level heavy metal contamination modulates the expression patterns of carbon- and nitrogen-cycling genes; whether such regulation varies significantly across different soil types and environmental conditions; and how interactions between Cd and other environmental factors influence the dynamics of carbon- and nitrogen-cycling genes. These limitations (e.g., reliance on DNA abundance rather than gene expression) might lead to an overestimation of functional potential, as they failed to distinguish between metabolic potential and actual activity under severe heavy metal stress. Therefore, these knowledge gaps highlight the need for future interdisciplinary research that integrates multi-omics approaches with long-term in situ monitoring to elucidate the impacts of severe heavy metal contamination on soil microbial functioning and biogeochemical cycling.

## 4. Materials and Methods

### 4.1. Greenhouse Pot Experiment and Sample Collection

The pot experiment was conducted in a sunlight greenhouse (day/night temperature, 32 °C/20 °C; relative humidity, 50–60%) at the Agricultural Water and Soil Environmental Field Science Research Station of the Chinese Academy of Agricultural Science (CAAS), located in Xinxiang of Henan Province, China (35°19′ N, 113°53′ E). The tested soil was taken from farmland topsoil on the outskirts of the city, and the properties were as follows: pH 8.24, electrical conductivity (EC) 439 μS·cm^−1^, organic matter (OM) 0.45%, total nitrogen (TN) 0.39 g·kg^−1^ and total phosphate (TP) 0.80 g·kg^−1^, and the total cadmium (Cd), lead (Pb), copper (Cu), and zinc (Zn) levels were 0.07 mg·kg^−1^, 7.99 mg·kg^−1^, 5.18 mg·kg^−1^, and 32.16 mg·kg^−1^.

After air-drying, each pot was filled with 7.5 kg of sieved soil. The Cd aqueous solution (CdCl_2_·2.5H_2_O) was evenly sprayed into the soil to homogenize well, and the treatments were designated as C5, C25, C50, C100, and C500, corresponding to 5 mg·kg^−1^, 25 mg·kg^−1^, 50 mg·kg^−1^, 100 mg·kg^−1^, and 500 mg·kg^−1^ of Cd^2+^ added to the soil, respectively. A treatment without Cd addition was used as the control (designated as C0). Each treatment was set to three replicates. The soil was equilibrated for one month. Ryegrass (*Lolium perenne* L.) seeds were surface sterilized by immersion in 75% ethanol (30 s) followed by soaking in 3% sodium hypochlorite (5 min), and then rinsed three times with sterile distilled water. The sterilized seeds were germinated in plastic cave dishes containing a common nursery substrate (peat: vermiculite = 2:1, *v*/*v*) and maintained in an artificial climate incubator (day/night temperatures of 25 °C/20 °C, a 16 h light/8 h dark photoperiod with a light intensity of 300 μmol m^−2^·s^−1^, and a relative humidity of 50–60%). After approximately two weeks, healthy and morphologically uniform seedlings were selected and transplanted into pots for the subsequent experiment. The fertilization levels were N (Urea) 200 mg·kg^−1^, P (NH_4_H_2_PO_4_) 150 mg·kg^−1^, and K (KCl) 150 mg·kg^−1^, respectively. After 90-day cultivation, rhizosphere soil samples were collected as follows: the entire root systems of all plants were gently excavated from the 0–20 cm depth, loosely attached soil was removed separately by shaking (approximately 20 g per replicate), and the soil tightly adhering to the roots was collected. The collected rhizosphere soil samples were immediately placed on ice, transported to the laboratory, and then stored at –80 °C after freeze-drying for subsequent DNA extraction and microbial community analysis. During the experimental period, plants in the C100 and C500 treatments exhibited visible symptoms of cadmium toxicity, including chlorosis, stunted growth, and reduced biomass, indicating severe stress and growth retardation at higher Cd concentrations.

### 4.2. Soil Physicochemical Properties

The air-dried and sieved soil samples were mixed with deionized water at a 1:2.5 (*w*/*v*) ratio, thoroughly shaken, and equilibrated for 30 min before measurement using a pH meter (Mettler Toledo) and conductivity meter (Eutech™ CON2700), respectively. The content of organic matter (OM) was determined by the method of “Soil testing Part 6: Method for determination of soil organic matter” (NY/T 1121.6-2006) [76]. The total nitrogen (TN) content was determined by the method of “Soil quality—Determination of total nitrogen—Modified Kjeldahl method” (HJ 717-2014) [77]. Total phosphorus (TP) was measured by the method of “Soil-Determination of Total Phosphorus by alkali fusion–Mo-Sb Anti spectrophotometric method” (HJ 632-2011) [78]. Total Cd content in the soil was analyzed using the method of “Soil quality-Determination of lead, cadmium-Graphite furnace atomic absorption spectrophotometry” (GB/T 17141-1997) [79].

### 4.3. Genomic DNA Extraction, Amplification, and Sequencing

The total genomic DNA from 18 rhizosphere soil samples was extracted using the FastDNA Spin Kit for Soil (MP Biomedicals, Santa Ana, CA, USA). The concentration and purity of the extracted DNA were determined by QuickDrop Spectrophotometer (Molecular Devices, LLC., San Jose, CA, USA). For bacteria, the V3-V4 region of the 16S rRNA gene was amplified using the primers 338F (5′-ACTCCTACGGGAGGCAGCAG-3′) and 806R (5′-GGACTACHVGGGTWTCTAAT-3′). The ITS region of fungi was amplified using the primer pairs ITS1 (5′-CTTGGTCATTTAGAGGAAGTAA-3′) and ITS2 (5′-GCTGCGTTCTTCAT-CGATGC-3′). PCR amplification was performed in a total volume of 20 µL containing 2 µL of 10× FastPfu Buffer, 0.8 µL of 5 µM of each forward and reverse primer, 0.2 µL of TransStart Fastpfu DNA Polymerase (TransGen Biotech Co., Ltd., Beijing, China), 0.2 µL of BSA, 10 ng of template DNA, and nuclease-free water for a total volume of 20 μL. The PCR reactions were performed in a GeneAmp^®^ 9700 thermal cycler (Applied Biosystems, Foster City, CA, USA) under the following reaction conditions: initial denaturation at 95 °C for 3 min; followed by 40 cycles of denaturation at 95 °C for 30 s, annealing at 55 °C for 30 s, and extension at 72 °C for 45 s; with a final extension at 72 °C for 10 min, 10 °C until halted by user. After PCR amplification, the purified amplicons were sequenced with the Illumina MiSeq PE300 platform (Illumina Inc., San Diego, CA, USA) according to the standard protocols by Majorbio Bio-Pharm Technology Co. Ltd. (Shanghai, China).

### 4.4. Quantification of Functional Genes

The *nirS*, *nirK*, and *nosZ* are key denitrification genes encoding nitrite and nitrous oxide reductases, serving as sensitive indicators of soil N transformation and N_2_O emission [80,81]. The *cbbL* gene, encoding the large subunit of ribulose-1,5-bisphosphate carboxylase/oxygenase (RubisCO) is a marker for autotrophic CO_2_ fixation [82]. These genes are commonly used as functional markers for soil C and N cycling under heavy metal stress. The primer pairs used in this study are listed in Table S1. For standard curve construction, the PCR products were purified with a PCR Purification Kit (Majorbio Sanger Bio-pharm Tech, Shanghai, China) and cloned using a pMD18-T Vector Cloning Kit (Takara Biotech, Dalian, China). The plasmid DNA from the cloned *E*. *coli* Top-10 was extracted using a Plasmid Prep Kit (Majorbio Sanger Bio-pharm Tech, Shanghai, China). The constructed plasmids were confirmed by sequencing, and then their OD260 values were determined using a NanoDrop UV-Vis spectrophotometer (BioTeke Co, Beijing, China). The gene copy number was calculated according to the plasmid concentration and the 10-fold serial dilution (10^−1^–10^−8^) of the plasmid DNA with known copy number was used to construct the standard curves. For all primer pairs amplifying gene fragments, the PCR mixture consisted of 10 μL 2× ChamQ SYBR Color qPCR Master Mix (Vazyme Biotech, Nanjing, China), 0.5 μL of each primer (5 μM), 1 μL of DNA and 8 μL of sterile ddH_2_O for a total volume 20 μL. All reactions were performed on a LineGene 9600 Plus thermal cycler (Bioer Technol, Hangzhou, China). The thermal cycling conditions consisted of an initial denaturation at 95 °C for 5 min, followed by 40 cycles of 30 s at 95 °C, 30 s at 50–57 °C, 40 s at 72 °C. To confirm the specificity of amplification and the absence of primer dimers, melt curve analysis was conducted for each assay. Three replicates were set for each run, and the PCR efficiencies (*E*) were 90–102% and correlation coefficients (*R*^2^) > 0.99.

### 4.5. Bioinformatics Analysis and Statistical Analysis

A detailed bioinformatics workflow of the sequencing data was performed on the I-Sanger Cloud Platform from Majorbio Bio-Pharm Technology Co. Ltd. (Shanghai, China) (https://cloud.majorbio.com). The processing of 16S rRNA gene and ITS sequences was performed following a standard procedure: (1) Taxonomic annotation and evaluation: All sequences were normalized to their lowest number of reads for further analysis. The representative OTUs of the 16S rRNA gene and ITS sequences were clustered at 97% similarity (Uparse version 7.0, http://drive5.com/uparse/, access on 15 August 2023), and α-diversity indices (Shannon, Simpson, Chao1, Coverage) were calculated using Mothur. (2) Microbial composition analysis: Venn diagrams were displayed by R package. Different taxonomic OTU analyses were performed on the QIIME platform (http://qiime.org/scripts/assign_taxonomy.html, access on 15 August 2023) at 97% similarity. Silva (Release 128, http://www.arb-silva.de) was used to analyze the microbial community composition for each sample. (3) Comparative analysis (*β*-diversity): Principal coordinate analysis (PCoA) based on Bray–Curtis distance was performed to investigate differences in microbial community structure. (4) Linear Discriminant Analysis Effect Size (LEfSe) was performed to identify bacterial taxa that were significantly different in relative abundance among treatment groups. The non-parametric Kruskal–Wallis rank-sum test was applied to detect taxa with significant differential abundance across all groups (*p* < 0.05). Linear discriminant analysis (LDA) was used to estimate the effect size of each differentially abundant taxon, and taxa with an LDA score > 3 were considered as potential biomarkers for specific treatments. (5) Correlation analysis between environmental factors and microbial community: Redundancy analysis (RDA) and heatmapping were conducted using R software (version 3.3.1) with “vegan” and “pheatmap” packages. The Mantel test was employed to evaluate the relationships between microbial community and soil environmental variables distance matrix. (6) Predictive functional analysis: FUNGuild analyses were used to predict the functions of fungi community. The functional prediction of bacteria community involved in biogeochemical cycles was carried out based on the artificial FAPROTAX database. The prediction of microbiome phenotypes were performed using BugBase (https://bugbase.cs.umn.edu/index.html). All raw data associated with this project has been deposited into the SRA database under the BioProject IDs PRJNA918828 (Bacteria) and PRJNA918779 (Fungi).

The data were analyzed using SPSS 20.0 software (SPSS, Chicago, IL, USA) and Excel 2007 and were expressed as the means ± SD. Significant difference was determined by a one-way analysis of variance; *p* < 0.05 was considered statistically significant.

## 5. Conclusions

Microorganisms survive in habitats shaped by diverse nutrient resources and environmental stresses and have evolved numerous mechanisms to resist and adapt to external pressures. The present study demonstrated that Cd stress induced a dose-dependent restructuring of the ryegrass rhizosphere microbiome, characterized by shifts in dominant taxa and functional profiles. Key bacterial taxa such as Bacteroidetes exhibited enhanced tolerance, while saprophytic fungi were enriched under high Cd conditions, collectively indicating an adaptive microbial response that likely supported host plant resilience. Soil properties (pH, EC, TN, and Cd) critically shaped community composition, with functional changes further implicating nutrient cycling and detoxification mechanisms. These findings provide systematic insights into the microbial adaptive strategies underpinning plant Cd tolerance, offering a theoretical basis for leveraging rhizosphere microbiomes in phytoremediation and sustainable agriculture. Despite the valuable insights gained from this study, several limitations should be acknowledged. The functional predictions derived from FAPROTAX and FUNGuild, as well as the gene abundances quantified by qPCR, represented the functional potential rather than the actual metabolic activities or gene expression levels. Therefore, future studies integrating metagenomics, metatranscriptomics, or metaproteomics are needed to directly validate the active expression of key ecological functions under field conditions.

## Figures and Tables

**Table 1:** The physicochemical properties of Cd-spiked soils.

Treatment	pH	EC (μS·cm^−1^)	OM (%)	TN (mg·g^−1^)	TP (mg·g^−1^)	Cd (mg·kg^−1^)
C0	8.25 ± 0.04 b	1017 ± 133 b	0.83 ± 0.03 a	0.21 ± 0.02 ab	0.45 ± 0.04 a	0.48 ± 0.08 f
C5	8.43 ± 0.05 a	1055 ± 67 b	0.84 ± 0.11 a	0.14 ± 0.05 bc	0.43 ± 0.01 a	16.93 ± 0.36 e
C25	8.47 ± 0.03 a	1244 ± 118 b	0.78 ± 0.04 a	0.12 ± 0.01 c	0.48 ± 0.03 a	55.09 ± 5.61 d
C50	8.50 ± 0.11 a	1117 ± 47 b	0.83 ± 0.12 a	0.15 ± 0.01 bc	0.43 ± 0.04 a	111.05 ± 5.47 c
C100	8.51 ± 0.08 a	1209 ± 241 b	0.85 ± 0.04 a	0.20 ± 0.05 ab	0.48 ± 0.02 a	181.71 ± 6.76 b
C500	8.10 ± 0.03 c	1873 ± 100 a	0.84 ± 0.02 a	0.25 ± 0.01 a	0.48 ± 0.01 a	1025.82 ± 51.25 a

Note: Results are expressed as the mean ± SD (*n* = 3); different lowercase letters indicate significant differences among the treatments (*p* < 0.05). Abbreviations: EC, electrical conductivity; OM, organic matter; TN, total nitrogen; TP, total phosphorus; Cd, total cadmium.

**Table 2:** *α* diversity indices of the microbial communities.

Treatments	Chao1		Coverage		Shannon		Simpson	
	Bacteria	Fungi	Bacteria	Fungi	Bacteria	Fungi	Bacteria	Fungi
C0	2320 ± 122 a	174 ± 18 b	0.986 ± 0.004 a	0.999 ± 0.000 a	5.71 ± 0.07 a	2.96 ± 0.22 a	0.011 ± 0.001 b	0.104 ± 0.022 b
C5	2268 ± 12 a	169 ± 6 b	0.985 ± 0.001 a	0.999 ± 0.000 a	5.83 ± 0.06 a	2.81 ± 0.13 a	0.009 ± 0.002 b	0.118 ± 0.019 b
C25	2206 ± 119 a	250 ± 75 b	0.987 ± 0.001 a	0.999 ± 0.000 a	5.38 ± 0.21 bc	2.89 ± 0.20 a	0.016 ± 0.005 a	0.104 ± 0.024 b
C50	2123 ± 82 a	214 ± 12 a	0.987 ± 0.001 a	0.999 ± 0.000 a	5.32 ± 0.13 c	2.81 ± 0.09 a	0.015 ± 0.003 a	0.115 ± 0.011 b
C100	2119 ± 62 a	269 ± 10 a	0.987 ± 0.002 a	0.999 ± 0.000 a	5.39 ± 0.07 bc	3.03 ± 0.06 a	0.015 ± 0.002 a	0.087 ± 0.008 b
C500	2131 ± 20 a	192 ± 8 b	0.986 ± 0.001 a	0.999 ± 0.000 a	5.62 ± 0.10 ab	2.58 ± 0.09 a	0.009 ± 0.001 b	0.157 ± 0.007 a

Note: Results are expressed as the mean ± SD (*n* = 3); different lowercase letters indicate significant differences between different treatments.

## Data Availability

The original contributions presented in this study are included in the article/Supplementary Material. Further inquiries can be directed to the corresponding author.

## Supplementary Materials

The following supporting information can be downloaded at https://www.mdpi.com/article/10.3390/plants15172634/s1.

## References

[1] Olaniran A.O., Balgobind A., Pillay B. (2011). Quantitative Assessment of the Toxic Effects of Heavy Metals on 1,2-dichloroethane Biodegradation in Co-contaminated Soil under Aerobic Condition. Chemosphere.

[2] Zhao F.J., Ma Y.B., Zhu Y.G., Tang Z., McGrath S.P. (2015). Soil Contamination in China: Current Status and Mitigation Strategies. Environ. Sci. Technol..

[3] Zhang X.Y., Zhong T.Y., Liu L., Ouyang X. (2015). Impact of Soil Heavy Metal Pollution on Food Safety in China. PLoS ONE.

[4] Chețan F., Moraru P.I., Rusu T., Șimon A., Dinca L., Murariu G. (2025). From Contamination to Mitigation: Addressing Cadmium Pollution in Agricultural Soils. Agriculture.

[5] Zhang S.Y., Tan L., Yuan L.W., Yang S., Wu Y.J., Jin J., Tong S.Q., Zhang L.C., Yan Y., Li C. (2026). Cadmium Contamination in Soil: Sources, Toxicological Impacts, Advanced Detection and Sustainable Remediation. Environ. Technol. Innov..

[6] Kim H.K., Jang T.I., Kim S.M., Park S.W. (2015). Impact of Domestic Wastewater Irrigation on Heavy Metal Contamination in Soil and Vegetables. Environ. Earth Sci..

[7] Chen W.P., Lu S.D., Peng C., Jiao W.T., Wang M. (2013). Accumulation of Cd in Agricultural Soil under Long-term Reclaimed Water Irrigation. Environ. Pollut..

[8] Lu X.Z., Gu A.Q., Huang C.L., Wei Y.C., Xu M.X., Yin H.Q., Hu X.F. (2021). Assessments of Heavy Metal Pollution of a Farmland in An Urban Area Based on the Environmental Geochemical Baselines. J. Soils Sediments.

[9] Cao X., Hu X., Efrizal E., Hayati I., Yang J., Tan C., Zhang M. (2024). Tradeoffs Among Yield, Cadmium Bioavailability, Nitrous Oxide Emission and Bacterial Community Stability: Effects of Iron-modified Woody Peat and Nitrification Inhibitors on Soil-Vegetable Systems. J. Environ. Manag..

[10] Papoyan A., Pineros M., Kochian L.V. (2007). Plant Cd^2+^ and Zn^2+^ Status Effects on Root and Shoot Heavy Metal Accumulation in *Thlaspi caerulescens*. New Phytol..

[11] Parmar P., Kumari N., Sharma V. (2013). Structural and Functional Alterations in Photosynthetic Apparatus of Plants under Cadmium Stress. Bot. Stud..

[12] Wang M., Chen W., Peng C. (2016). Risk Assessment of Cd Polluted Paddy Soils in the Industrial and Township Areas in Hunan, Southern China. Chemosphere.

[13] Wu H., Liao Q., Chillrud S., Yang Q., Huang L., Bi J., Yan B. (2016). Environmental Exposure to Cadmium: Health Risk Assessment and Its Associations with Hypertension and Impaired Kidney Function. Sci. Rep..

[14] Zhang X., Xia H., Li Z.A., Zhuang P., Gao B. (2011). Identification of a New Potential Cd-hyperaccumulator *Solanum photeinocarpum* by Soil Seed Bank-metal Concentration Gradient Method. J. Hazard. Mater..

[15] Sun R.L., Zhou Q.X., Jin C.X. (2006). Cadmium Accumulation in Relation to Organic Acids in Leaves of *Solanum nigrum* L. as a Newly Found Cadmium Hyperaccumulator. Plant Soil.

[16] Luo H.J., Li H.Y., Zhang X.Z., Fu J.M. (2011). Antioxidant Responses and Gene Expression in Perennial Ryegrass (*Lolium perenne* L.) under Cadmium Stress. Ecotoxicology.

[17] Rascio N., Navari-Izzo F. (2011). Heavy Metal Hyperaccumulating Plants: How and Why do They Do It? And What Makes Them so Interesting?. Plant Sci..

[18] Lou Y.H., Luo H.J., Hu T., Li H., Fu J. (2013). Toxic Effects, Uptake, and Translocation of Cd and Pb in Perennial Ryegrass. Ecotoxicology.

[19] Song N.N., Wang F.L., Ma Y.B., Tang S.R. (2015). Using DGT to Assess Cadmium Bioavailability to Ryegrass as Influenced by Soil Properties. Pedosphere.

[20] Moreno J.L., Sanchez-Marín A., Hernández T., García C. (2006). Effect of Cadmium on Microbial Activity and a Ryegrass Crop in Two Semiarid Soils. Environ. Manag..

[21] Xie H., Ma Y., Wang Y., Sun F.X., Liu R.Y., Liu X., Xu Y.X. (2021). Biological Response and Phytoremediation of Perennial Ryegrass to Halogenated Flame Retardants and Cd in Contaminated Soils. J. Environ. Chem. Eng..

[22] He S.Y., Li Y., Guo H.H., Lu L., Yang C.P. (2020). Combined Effect of Ryegrass and *Hyphomicrobium* sp. GHH on the Remediation of EE2-Cd Co-contaminated Soil. J. Soils Sediments.

[23] Hinsinger P., Plassard C., Jaillard B. (2006). Rhizosphere: A New Frontier for Soil Biogeochemistry. ChemInform.

[24] Wood J.L., Zhang C., Mathews E.R., Tang C., Franks A.E. (2016). Microbial Community Dynamics in the Rhizosphere of a Cadmium Hyper-accumulator. Sci. Rep..

[25] Niu H., Leng Y.F., Li X.C., Yu Q., Wu H., Gong J.C., Li H.L., Chen K. (2021). Behaviors of Cadmium in Rhizosphere Soils and Its Interaction with Microbiome Communities in Phytoremediation. Chemosphere.

[26] Khan M.F. (2025). Microbial Remediation of Agrochemical-Contaminated Soils: Enzymatic Mechanisms, Quorum Sensing, and Emerging Opportunities. Integr. Environ. Assess. Manag..

[27] Song L., Pan Z.Z., Dai Y., Chen L., Zhang L., Liao Q., Yu X., Guo H., Zhou G. (2020). Characterization and Comparison of the Bacterial Communities of Rhizosphere and Bulk Soils from Cadmium-polluted Wheat Fields. PeerJ.

[28] Feng G., Xie T., Wang X., Bai J., Tang L., Zhao H., Wei W., Wang M., Zhao Y. (2018). Metagenomic Analysis of Microbial Community and Function Involved in Cd-contaminated Soil. BMC Microbiol..

[29] Khan M.A., Khan S., Khan A., Alam M. (2017). Soil Contamination with Cadmium, Consequences and Remediation using Organic Amendments. Sci. Total Environ..

[30] Farooqi A., Haq E.U., Johansen A., Ellegaard-Jensen L., Iqbal M., Yousaf S., Lackner M. (2025). Plant Growth-promoting Bacteria and Nanomaterials Synergism to Enhance *Lolium perenne* Growth and Phytoremediation in Cadmium-contaminated Soil. Chem. Eng. J. Adv..

[31] Kou B., Yu T.Q., Tang J., Zhu X., Yuan Y., Tan W. (2024). Kitchen Compost-derived Humic Acid Application Promotes Ryegrass Growth and Enhances the Accumulation of Cd: An Analysis of the Soil Microenvironment and Rhizosphere Functional Microbes. Sci. Total Environ..

[32] Ma H., Wei M., Wang Z., Hou S., Li X., Xu H. (2020). Bioremediation of Cadmium Polluted Soil using a Novel Cadmium Immobilizing Plant Growth Promotion Strain *Bacillus* sp. TZ5 Loaded on Biochar. J. Hazard. Mater..

[33] Mathivanan K., Chandirika J.U., Vinothkanna A., Yin H., Liu X., Meng D. (2021). Bacterial Adaptive Strategies to Cope with Metal Toxicity in the Contaminated Environment—A review. Ecotoxicol. Environ. Saf..

[34] Liao M., Xie M.X. (2007). Effect of Heavy Metals on Substrate Utilization Pattern, Biomass, and Activity of Microbial Communities in a Reclaimed Mining Wasteland of Red Soil Area. Ecotoxicol. Environ. Saf..

[35] Cui Y.X., Wang X., Wang X.X., Zhang X.C., Fang L.C. (2021). Evaluation Methods of Heavy Metal Pollution in Soils based on Enzyme Activities: A Review. Soil Ecol. Lett..

[36] Yin K., Wang Q.N., Lv M., Chen L.X. (2019). Microorganism Remediation Strategies Towards Heavy Metals. Chem. Eng. J..

[37] Gao Y., Miao C.Y., Xia J., Mao L., Wang Y.F., Zhou P. (2012). Plant Diversity Reduces the Effect of Multiple Heavy Metal Pollution on Soil Enzyme Activities and Microbial Community Structure. Front. Environ. Sci. Eng..

[38] Thijs S., Langill T., Vangronsveld J. (2017). The Bacterial and Fungal Microbiota of Hyperaccumulator Plants: Small Organisms, Large Influence. Adv. Bot. Res..

[39] Chen X., Zhao Y., Zhao X., Wu J., Zhu L., Zhang X., Wei Z., Liu Y., He P. (2020). Selective Pressures of Heavy Metals on Microbial Community Determine Microbial Functional Roles during Composting: Sensitive, Resistant and Actor. J. Hazard. Mater..

[40] Lazzaro A., Widmer F., Sperisen C., Frey B. (2008). Identification of Dominant Bacterial Phylotypes in a Cadmium-treated Forest Soil. FEMS Microbiol. Ecol..

[41] Abid A.A., Zhang G., He D., Wang H., Batool I., Di H., Zhang Q. (2022). Combined Effects of *Bacillus* sp. M6 Strain and *Sedum alfredii* on Rhizosphere Community and Bioremediation of Cadmium Polluted Soils. Front. Plant Sci..

[42] Wang X., Ya T., Zhang M., Liu L., Hou P., Lu S. (2019). Cadmium (II) Alters the Microbial Community Structure and Molecular Ecological Network in Activated Sludge System. Environ. Pollut..

[43] Ding Z.L., Wu J.P., You A.Q., Huang B.Q., Cao C.G. (2017). Effects of Heavy Metals on Soil Microbial Community Structure and Diversity in the Rice (*Oryza sativa* L. subsp. Japonica, Food Crops Institute of Jiangsu Academy of Agricultural Sciences) Rhizosphere. Soil Sci. Plant Nutr..

[44] Wang T.J., Su N.N., Lei P., Qiu M.Y., Chen Z.J., Yao L.G., Han H. (2019). Community Structure of Heavy Metal Immobilized Bacteria in the Lettuce (*Lactuca sativa* L.) Rhizosphere in Soil Polluted by Heavy Metals and Its Effects on Reducing Heavy Metal Accumulation in Lettuce. Environ. Sci..

[45] Deng L., Zeng G., Fan C., Lu L., Chen X., Chen M., Wu H., He X., He Y. (2015). Response of Rhizosphere Microbial Community Structure and Diversity to Heavy Metal Co-pollution in Arable Soil. Appl. Microbiol. Biotechnol..

[46] Gupta P., Diwan B. (2017). Bacterial Exopolysaccharide Mediated Heavy Metal Removal: A Review on Biosynthesis, Mechanism and Remediation Strategies. Biotechnol. Rep..

[47] Zhang B., Xu J., Sun M., Yu P., Ma Y., Xie L., Chen L. (2023). Comparative Secretomic and Proteomic Analysis Reveal Multiple Defensive Strategies Developed by *Vibrio cholerae* Against the Heavy Metal (Cd^2+^, Ni^2+^, Pb^2+^, and Zn^2+^) Stresses. Front. Microbiol..

[48] Wu C.H., Wood T.K., Mulchandani A., Chen W. (2006). Engineering Plant-microbe Symbiosis for Rhizoremediation of Heavy Metals. Appl. Environ. Microbiol..

[49] Mohammad A., Mittra B. (2013). Effects of Inoculation with Stress-adapted Arbuscular Mycorrhizal Fungus *Glomus deserticola* on Growth of *Solanum melogena* L. and *Sorghum sudanese* Staph. Seedlings under Salinity and Heavy Metal Stress Conditions. Arch. Agron. Soil. Sci..

[50] Philippot L., Raaijmakers J.M., Lemanceau P., van der Putten W.H. (2013). Going Back to the Roots: The Microbial Ecology of the Rhizosphere. Nat. Rev. Microbiol..

[51] Chen J.H., He F., Zhang X.H., Sun X., Zheng J., Zheng J. (2014). Heavy Metal Pollution Decreases Microbial Abundance, Diversity and Activity within Particle-size Fractions of a Paddy Soil. FEMS Microbiol. Ecol..

[52] Xie Y., Fan J., Zhu W., Amombo E., Lou Y., Chen L., Fu J. (2016). Effect of Heavy Metals Pollution on Soil Microbial Diversity and Bermuda Grass Genetic Variation. Front. Plant Sci..

[53] Zhao X., Huang J., Lu J., Sun Y. (2019). Study on the Influence of Soil Microbial Community on the Long-term Heavy Metal Pollution of Different Land Use Types and Depth Layers in Mine. Ecotoxicol. Environ. Saf..

[54] Zeng X.Y., Li S.W., Leng Y., Kang X.H. (2020). Structural and Functional Responses of Bacterial and Fungal Communities to Multiple Heavy Metal Exposure in Arid Loess. Sci. Total Environ..

[55] Hu X., Wang J., Lv Y., Liu X., Zhong J., Cui X., Zhang M., Ma D., Yan X., Zhu X. (2021). Effects of Heavy Metals/Metalloids and Soil Properties on Microbial Communities in Farmland in the Vicinity of a Metals Smelter. Front. Microbiol..

[56] Duan C., Wang Y., Wang Q., Ju W., Zhang Z., Cui Y., Beiyuan J., Fan Q., Wei S., Li S. (2022). Microbial Metabolic Limitation of Rhizosphere under Heavy Metal Stress: Evidence from Soil Ecoenzymatic Stoichiometry. Environ. Pollut..

[57] Wu Y., Wang H., Peng L., Zhao H., Zhang Q., Tao Q., Tang X., Huang R., Li B., Wang C. (2024). Root-Soil-Microbiome Interaction in the Rhizosphere of Masson Pine (*Pinus massoniana*) under Different Levels of Heavy Metal Pollution. Ecotoxicol. Environ. Saf..

[58] He X., Xiao X., Wei W., Li L., Zhao Y., Zhang N., Wang M. (2024). Soil Rare Microorganisms Mediated the Plant Cadmium Uptake: The Central Role of Protists. Sci. Total Environ..

[59] Duan L.Y., Zhang Y., Li Y.Y., Li X.Q., Liu Y.Q., Li B.L., Ding C.Y., Ren X.M., Duan P.F., Han H. (2024). Effects of Combined Microplastic and Cadmium Pollution on Sorghum Growth, Cd Accumulation, and Rhizosphere Microbial Functions. Ecotoxicol. Environ. Saf..

[60] Njoku K.L., Asunmo M.O., Ude E.O., Adesuyi A.A., Oyelami A.O. (2020). The Molecular Study of Microbial and Functional Diversity of Resistant Microbes in Heavy Metal Contaminated Soil. Environ. Technol. Innov..

[61] Li S., Li G., Huang X., Chen Y., Lv C., Bai L., Zhang K., He H., Dai J. (2023). Cultivar-specific Response of Rhizosphere Bacterial Community to Uptake of Cadmium and Mineral Elements in Rice (*Oryza sativa* L.). Ecotoxicol. Environ. Saf..

[62] Li Q., Xing Y., Huang B., Chen X., Ji L., Fu X., Li T., Wang J., Chen G., Zhang Q. (2022). Rhizospheric Mechanisms of *Bacillus subtilis* Bioaugmentation-assisted Phytostabilization of Cadmium-contaminated Soil. Sci. Total Environ..

[63] Ji J.H., Zhong Y.Y., Xiao M.L., Wang X.T., Hu Z.E., Zhan M.J., Ding J.N., Zhu Z.K., Ge T.D. (2025). Synergistic Effect of Microplastics and Cadmium on Microbial Community and Functional Taxa in Wheat Rhizosphere Soil. Soil Ecol. Lett..

[64] Chen Y.P., Liu Q., Liu Y.J., Jia F.A., He X.H. (2014). Responses of Soil Microbial Activity to Cadmium Pollution and Elevated CO_2_. Sci. Rep..

[65] Li M.Y., Ren L., Zhang J., Luo L., Qin P., Zhou Y., Huang C., Tang J., Huang H., Chen A. (2019). Population Characteristics and Influential Factors of Nitrogen Cycling Functional Genes in Heavy Metal Contaminated Soil Remediated by Biochar and Compost. Sci. Total Environ..

[66] Li H.F., Abbas T., Cai M., Zhang Q., Wang J., Li Y., Di H., Tahir M. (2021). Cd Bioavailability and Nitrogen Cycling Microbes Interaction Affected by Mixed Amendments under Paddy-pak choi Continued Planting. Environ. Pollut..

[67] Zhang P.P., Zhang Y.H., Pang W.B., Alonazi M., Alwathnani H., Rensing C., Xie R., Zhang T. (2024). *Cenococcum geophilum* Impedes Cadmium Toxicity in *Pinus massoniana* by Modulating Nitrogen Metabolism. Sci. Total Environ..

[68] Yang L., Yang Z., Ma X., Wang J., Li J., Lin Y.B., Fan M.C., Shangguan Z.P. (2023). Response of Rare and Abundant Rhizosphere Microbial Communities to Inoculated Rhizobium in Cadmium-contaminated Soil Phytoremediation. Rhizosphere.

[69] Afzal M., Yu M., Tang C., Zhang L., Muhammad N., Zhao H., Feng J., Yu L., Xu J. (2019). The Negative Impact of Cadmium on Nitrogen Transformation Processes in a Paddy Soil is Greater under Non-flooding than Flooding Conditions. Environ. Int..

[70] Yang Y.J., Liu H.X., Wang H., Li C.J., Lv J.L. (2024). Strategies of Soil Microbial N-cycling in Different Cadmium Contaminated Soil with Wheat Straw Return. Ecotoxicol. Environ. Saf..

[71] Ma L., Li Z.X., Liu G.H., Liu W.Z. (2023). Low-level Cadmium Alleviates the Disturbance of Doxycycline on Nitrogen Removal and N_2_O Emissions in Ditch Wetlands by Altering Microbial Community and Enzymatic Activity. J. Clean. Product..

[72] Elrys A.S., Wen Y., Feng D., El-Mekkawy R.M., Kong M., Qin X., Lu Q., Dan X., Zhu Q., Tang S. (2025). Cadmium Inhibits Carbon and Nitrogen Cycling through Soil Microbial Biomass and Reduces Soil Nitrogen Availability. J. Hazard. Mater..

[73] Zhao H.C., Lin J.H., Wang X.H., Shi J., Dahlgren R.A., Xu J. (2021). Dynamics of Soil Microbial N-Cycling Strategies in Response to Cadmium Stress. Environ. Sci. Technol..

[74] Xiao Z., Duan C., Li S., Chen J., Peng C., Che R., Liu C., Huang Y., Mei R., Xu L. (2023). The Microbial Mechanisms by Which Long-term Heavy Metal Contamination Affects Soil Organic Carbon Levels. Chemosphere.

[75] Xu M.Z., Cui Y.X., Beiyuan J.Z., Wang X., Duan C.J., Fang L.C. (2021). Heavy Metal Pollution Increases Soil Microbial Carbon Limitation: Evidence from Ecological Enzyme Stoichiometry. Soil Ecol. Lett..

[76] (2006). Soil Testing Part 6: Method for Determination of Soil Organic Matter.

[77] (2014). Soil Quality—Determination of total Nitrogen—Modified Kjeldahl Method.

[78] (2011). Soil-Determination of Total Phosphorus by Alkali Fusion–Mo-Sb Anti Spectrophotometric Method.

[79] (1997). Soil Quality-Determination of Lead, Cadmium-Graphite Furnace Atomic Absorption Spec-trophotometry.

[80] Throbäck I.N., Enwall K., Jarvis A., Hallin S. (2004). Reassessing PCR Primers Targeting *nirS*, *nirK* and *nosZ* Genes for Community Surveys of Denitrifying Bacteria with DGGE. FEMS Microbiol. Ecol..

[81] Henry S., Bru D., Stres B., Hallet S., Philippot L. (2006). Quantitative Detection of the *nosZ* Gene, Encoding Nitrous Oxide Reductase, and Comparison of the Abundances of 16S rRNA, *narG*, *nirK*, and *nosZ* Genes in Soils. Appl. Environ. Microbiol..

[82] Nanba K., King G.M., Dunfield K. (2004). Analysis of Facultative Lithotroph Distribution and Diversity on Volcanic Deposits by Use of the Large Subunit of Ribulose 1,5-Bisphosphate Carboxylase/oxygenase. Appl. Environ. Microbiol..

