# Supplementary material for: Integrated Adaptive Responses of Rhizosphere Microbial Communities and Functional Genes to Cadmium Stress in Ryegrass (Lolium perenne L.)
Source: Plants (Basel). 2026 Aug 28;15(17):2634. doi: 10.3390/plants15172634 (PMC13567738; doi:10.3390/plants15172634)
Supplement: Supplementary file 1 [file plants-15-02634-s001.zip › 20260701 Table SI.pdf]

Table S1. Selected primer sets for qPCR.

Gene	Sequence (5'-3')	length of amplicon	Reference
nirK	nirK-F: ATCATGGTSCTGCCGCG<br>nirK-R: GCCTCGATCAGRTTGTGGTT	473	[1]
nirS	cd3AF: GTSAACGTSAAGGARACSGG<br>R3cdR: GASTTCGGRTGSGTCTTGA	410	[1]
nosZ	NosZ2F: CGCRACGGCAASAAGGTSMSSGT<br>NosZ2R: CAKRTGCAKSGCRTGGCAGAA	267	[2]
cbbL	K2F: ACCAYCAAGCCSAAGCTSGG<br>V2R: GCCTTCSAGCTTGCCSACCRC	492	[3]

1. Throbäck, I.N.; Enwall, K.; Jarvis, A.; Hallin, S. Reassessing PCR primers targeting *nirS*, *nirK* and *nosZ* genes for community surveys of denitrifying bacteria with DGGE. *FEMS Microbiol. Ecol*. **2004**, *49*, 401-417.
2. Henry, S.; Bru, D.; Stres, B.; Hallet, S.; Philippot, L. Quantitative detection of the *nosZ* Gene, encoding nitrous oxide reductase, and comparison of the abundances of 16S rRNA, *narG*, *nirK*, and *nosZ* genes in soils. *Appl. Environ. Microbiol*. **2006**, *72(8)*, 5181-5189.
3. Nanba, K.; King, G.M.; Dunfield, K. Analysis of facultative lithotroph distribution and diversity on volcanic deposits by use of the large subunit of ribulose 1,5-bisphosphate carboxylase/oxygenase. *Appl. Environ. Microbiol*. **2004**, *70(4)*, 2245-2253.

Table S2. Pearson's correlation coefficient of soil properties and functional gene abundances.

	pH	EC	OM	TP	TN	Cd	*nirK*	*nirS*	*nosZ*
EC	**-0.730****								
OM	0.207	0.002							
TP	**-0.639***	0.512	-0.346						
TN	**-0.784****	**0.612***	0.152	**0.603***					
Cd	**-0.712****	**0.964****	0.020	0.533	**0.646***				
nirK	0.019	0.278	-0.022	0.119	0.255	0.288			
nirS	-0.494	**0.911****	-0.042	0.492	0.499	**0.923****	0.543		
nosZ	-0.186	0.201	-0.190	0.232	0.399	0.230	**0.879****	0.389	
cbbL	-0.350	**0.840****	0.009	0.468	0.414	**0.831****	0.575	**0.956****	0.328

Correlation coefficients significant at the 0.05 level are marked with one asterisk, while those with a *P* value of 0.01 are identified with two asterisks.
