# Supplementary material for: Integrated Adaptive Responses of Rhizosphere Microbial Communities and Functional Genes to Cadmium Stress in Ryegrass (Lolium perenne L.)
Source: Plants (Basel). 2026 Aug 28;15(17):2634. doi: 10.3390/plants15172634 (PMC13567738; doi:10.3390/plants15172634)
Supplement: Supplementary file 1 [file plants-15-02634-s001.zip › 20260701 Fig SI.pdf]

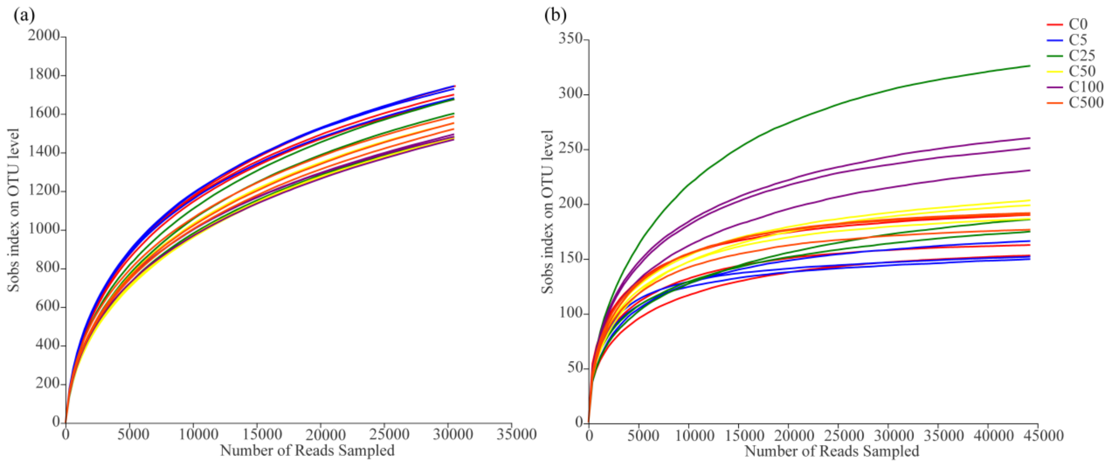

Figure S1. Rarefaction curves of the microbial communities.

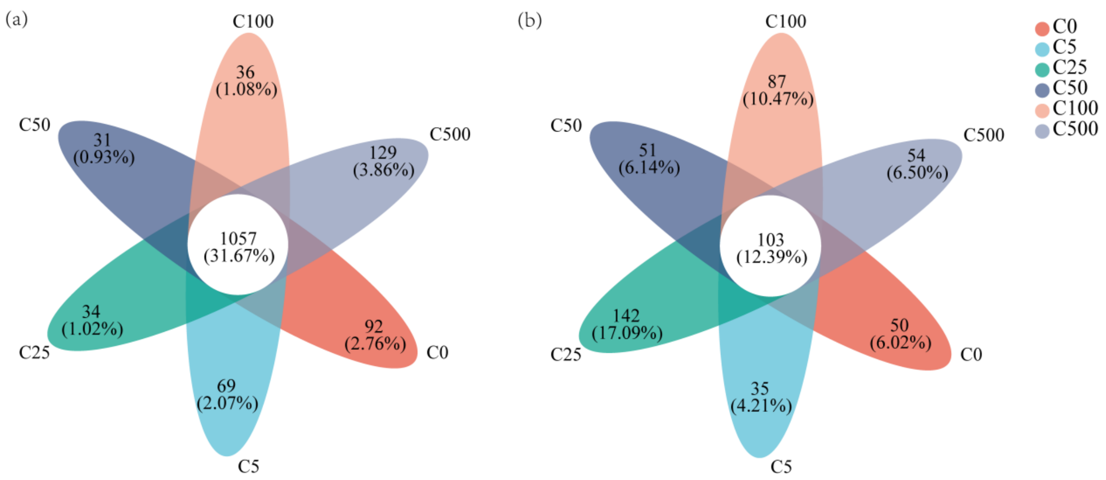

Figure S2. Venn diagrams showing shared and unique OTUs at 97% identity in bacterial (a) and fungal (b) communities among different groups.

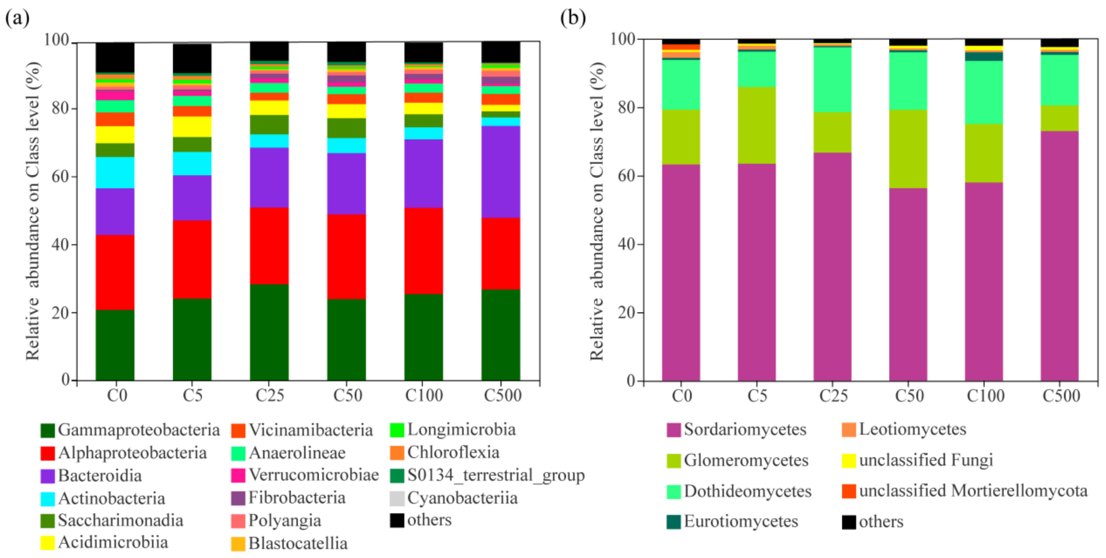

Figure S3. Soil bacterial (a) and fungal (b) community composition and relative abundance differences at the class levels.

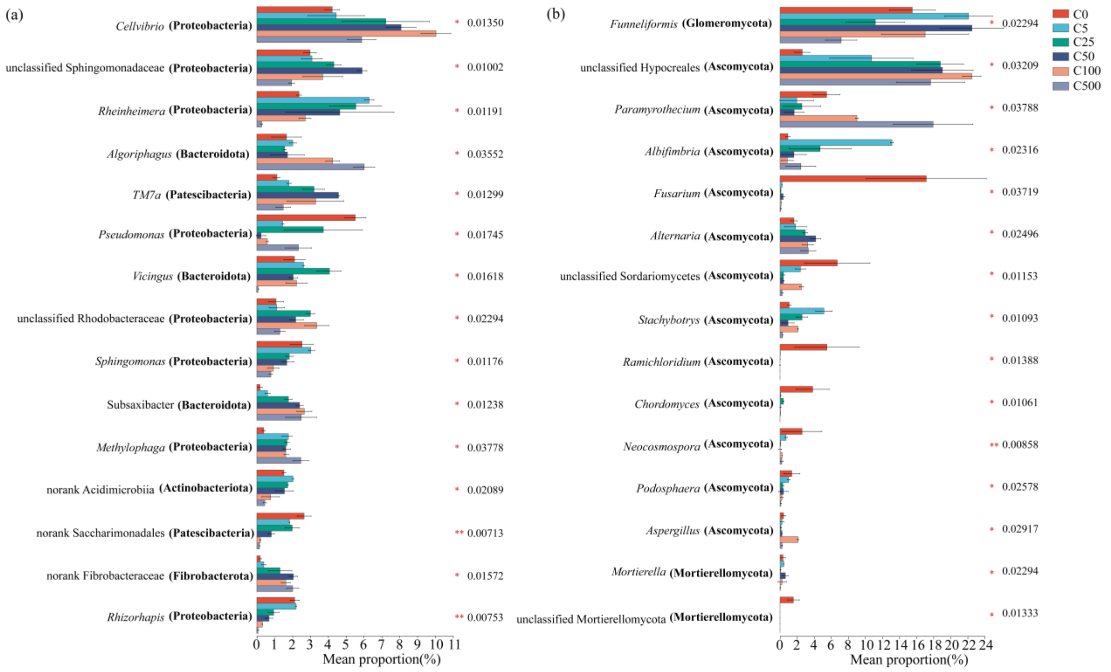

Figure S4. Soil bacterial (a) and fungal (b) relative abundance differences at genus level (* $0.01 < P \leq 0.05$, ** $0.001 < P \leq 0.01$).

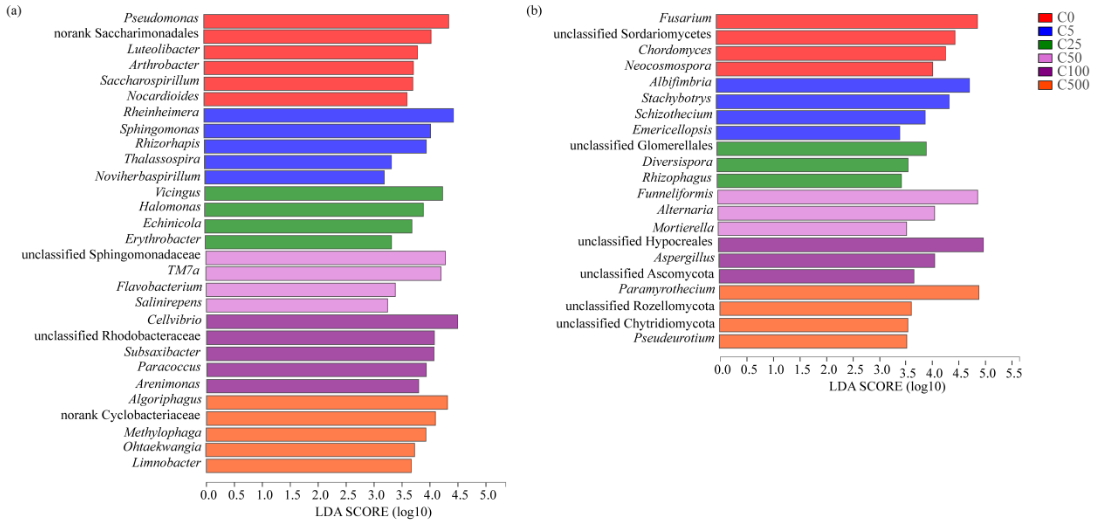

Figure S5. Indicator bacteria (a) and fungi (b) with LDA scores of 2 or greater in microbial communities associated with soil treated by different Cd concentration.

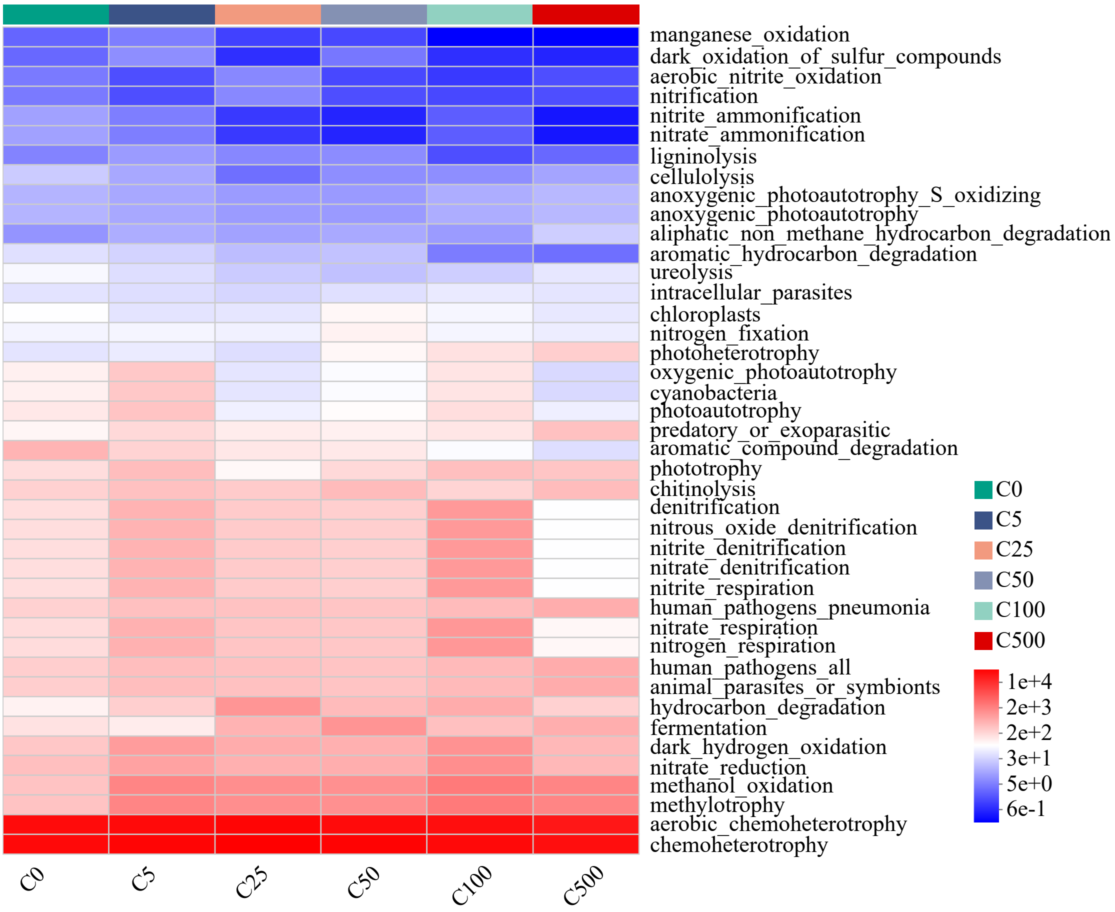

Figure S6. Heatmap of the FAPROTAX analysis reveals the differences in functional potential.

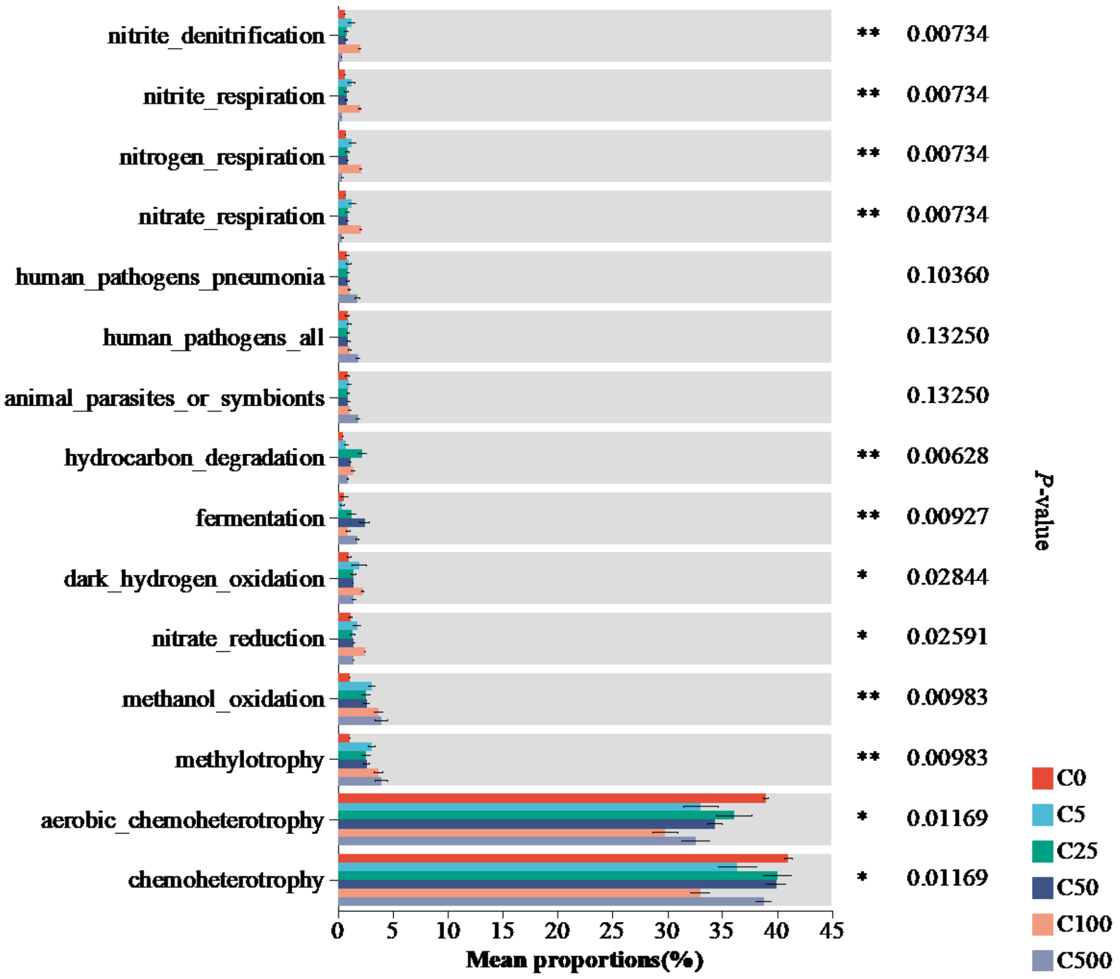

Figure S7. Histogram of the FAPROTAX analysis reveals the functional differences among treatments (0.01 < $P \leq 0.05$, ** 0.001 < $P \leq 0.01$).

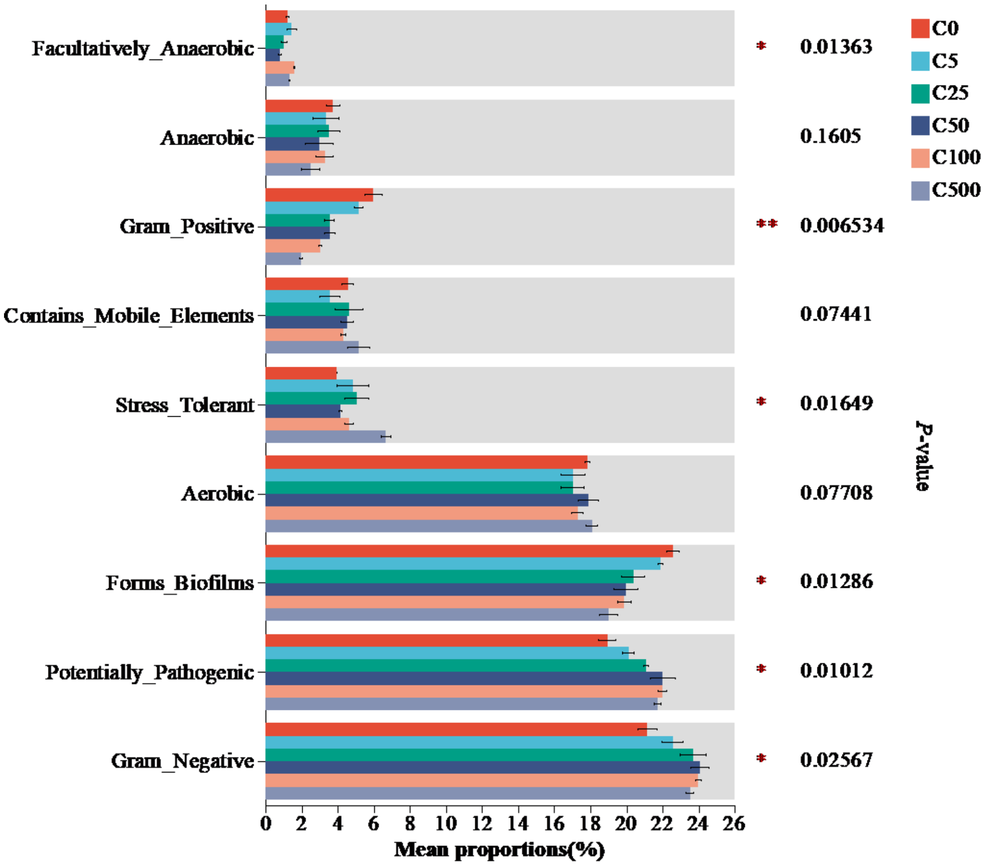

Figure S8. BugBase-based phenotypic predictions.

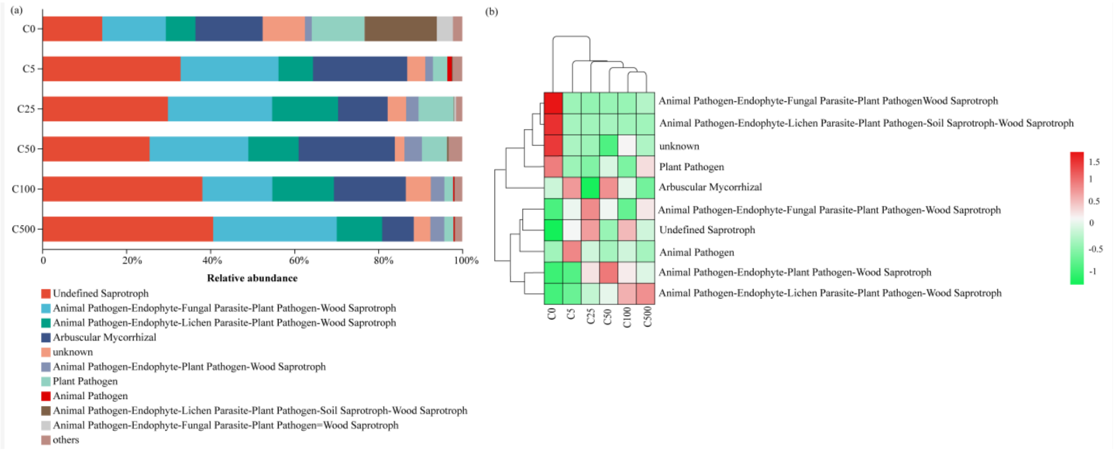

Figure S9. Variations in composition of fungal functional groups inferred by FUNGuild.
